# Effects of different types of low‐intensity management on plant‐pollinator interactions in Estonian grasslands

**DOI:** 10.1002/ece3.8325

**Published:** 2021-11-22

**Authors:** Elena Motivans Švara, Valentin Ştefan, Esther Sossai, Reinart Feldmann, Dianne Joy Aguilon, Anna Bontsutsnaja, Anna E‐Vojtkó, Isabel C. Kilian, Piret Lang, Marilin Mõtlep, Elisabeth Prangel, Mari‐Liis Viljur, Tiffany M. Knight, Lena Neuenkamp

**Affiliations:** ^1^ Department of Community Ecology Helmholtz Centre for Environmental Research – UFZ Halle (Saale) Germany; ^2^ German Centre for Integrative Biodiversity Research (iDiv) Halle‐Jena‐Leipzig Leipzig Germany; ^3^ Institute of Biology Martin Luther University Halle‐Wittenberg Halle (Saale) Germany; ^4^ Doctoral School of Environmental Sciences University of Szeged Szeged Hungary; ^5^ Department of Forest Biological Sciences College of Forestry and Natural Resources University of the Philippines Los Baños Laguna Philippines; ^6^ Department of Ecology University of Szeged Szeged Hungary; ^7^ Institute of Agricultural and Environmental Sciences Estonian University of Life Sciences Tartu Estonia; ^8^ Department of Botany Faculty of Science University of South Bohemia České Budějovice Czech Republic; ^9^ Institute of Botany Czech Academy of Sciences Třeboň Czech Republic; ^10^ Zoological Research Museum Alexander Koenig Leibniz Institute for Animal Biodiversity Bonn Germany; ^11^ Agroecology and Organic Farming Group (INRES‐AOL) University of Bonn Bonn Germany; ^12^ Department of Botany Institute of Ecology and Earth Sciences University of Tartu Tartu Estonia; ^13^ Department of Zoology Institute of Ecology and Earth Sciences University of Tartu Tartu Estonia; ^14^ Institute of Plant Sciences University of Bern Bern Switzerland

**Keywords:** biodiversity, conservation, land use, plant−pollinator networks, seminatural grasslands

## Abstract

In the face of global pollinator decline, extensively managed grasslands play an important role in supporting stable pollinator communities. However, different types of extensive management may promote particular plant species and thus particular functional traits. As the functional traits of flowering plant species (e.g., flower size and shape) in a habitat help determine the identity and frequency of pollinator visitors, they can also influence the structures of plant−pollinator interaction networks (i.e., pollination networks). The aim of this study was to examine how the type of low‐intensity traditional management influences plant and pollinator composition, the structure of plant−pollinator interactions, and their mediation by floral and insect functional traits. Specifically, we compared mown wooded meadows to grazed alvar pastures in western Estonia. We found that both management types fostered equal diversity of plants and pollinators, and overlapping, though still distinct, plant and pollinator compositions. Wooded meadow pollination networks had significantly higher connectance and specialization, while alvar pasture networks achieved higher interaction diversity at a standardized sampling of interactions. Pollinators with small body sizes and short proboscis lengths were more specialized in their preference for particular plant species and the specialization of individual pollinators was higher in alvar pastures than in wooded meadows. All in all, the two management types promoted diverse plant and pollinator communities, which enabled the development of equally even and nested pollination networks. The same generalist plant and pollinator species were important for the pollination networks of both wooded meadows and alvar pastures; however, they were complemented by management‐specific species, which accounted for differences in network structure. Therefore, the implementation of both management types in the same landscape helps to maintain high species and interaction diversity.

## Graphical abstract

We studied whether mown and grazed grasslands have different pollination network structures. We found higher network‐level connectance and specialization in mown sites and higher network‐level interaction diversity in the grazed sites. The same generalist plants and pollinators were important for the pollination networks of both mown and grazed sites; however, they were complemented by management‐specific species, which accounted for differences in network structure.

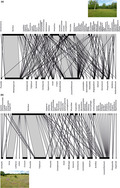

## 1 INTRODUCTION

Pollinators are declining globally due to multiple drivers, including habitat loss and degradation (Potts et al., 2010). This decline increases the importance of managing habitats so that they provide sufficient floral resources to support healthy pollinator communities. In Europe, seminatural, extensively managed grasslands play an important role in supporting rich pollinator communities that provide pollination services to the surrounding areas (Garibaldi et al., 2011; Jakobsson & Ågren, 2014; Seibold et al., 2019). However, different types of extensive management may promote particular plant species and thus functional traits (Tälle et al., 2016). As the functional traits of flowering plant species (e.g., flower size and shape) in a habitat help determine the identity and frequency of pollinator visitors (Klumpers et al., 2019; Stang et al., 2007), they can also influence the structures of plant−pollinator interaction networks (Fantinato et al., 2019). Understanding these effects can help inform the management of seminatural grasslands to support diverse plant−pollinator interactions and thereby the pollination of wild plants and crops.

Seminatural grasslands are some of the most plant and pollinator diverse ecosystems in Europe (Wilson et al., 2012). Therefore, conservation of these areas is critical to the preservation of these species and the pollination interactions between them. These ecosystems are maintained by extensive (i.e., traditional) management comprising of regular grazing or mowing, both of which foster pollinator (Lázaro, Tscheulin, Devalez, Nakas, & Petanidou, 2016; Lázaro, Tscheulin, Devalez, Nakas, Stefanaki, et al., 2016; Söderström et al., 2001; Weiner et al., 2011) and herbaceous plant species diversity (Fantinato et al., 2019; Fontana et al., 2014; Lázaro, Tscheulin, Devalez, Nakas, & Petanidou, 2016; Lázaro, Tscheulin, Devalez, Nakas, Stefanaki, et al., 2016; Römermann et al., 2009). However, the two management types have been shown to support different plant species compositions (Fantinato et al., 2019; Fontana et al., 2014). Grazing is a selective management type as grazers selectively remove functional groups of plant species (Oleques et al., 2019) and promote resistant, ruderal species (Vázquez & Simberloff, 2003; Yoshihara et al., 2008). Mowing, on the other hand, is an unselective management type that involves the periodic removal of all plant material, which prevents the spread of dominant plant species (Catorci et al., 2011). The response of pollinators to these management types has been found to be species and habitat specific, for example, bee abundance and richness were found to be affected by grazing (Lázaro, Tscheulin, Devalez, Nakas, & Petanidou, 2016, Vulliamy et al., 2006) and yet were not affected by grazing in another instance (Kimoto et al., 2012).

As grazing and mowing shape species composition, they subsequently also affect the structure of plant−pollinator interaction networks (henceforth referred to as pollination networks). When mowing and grazing are directly compared, extensive management by mowing has been shown to produce smaller networks with higher connectance than grazing (Kovács‐Hostyánszki et al., 2019). When looking at species roles in the networks, plant and pollinator specialization (d’) remained the same between the two management types (Fantinato et al., 2019). However, pollinators shared between the two management types had higher species strength than pollinators only found in mown or grazed sites, while shared plant species had lower species strength than plants only found in mown or grazed sites (Fantinato et al., 2019). Moreover, increased vegetation complexity has been shown to increase pollination network nestedness (Moreira et al., 2015). Thus, extensive land‐use management type is likely a very important driver of pollination network structure, but there are currently very few comparative studies for clear synthesis on the direction and magnitude of these effects.

Examining how functional traits change between management types and relate to network structure can provide a more mechanistic understanding for changes in network structure. Plant vegetative traits, such as plant height, leaf dry mass, life history, and specific leaf area, have been well‐studied and linked to grazing and other land‐use management (Díaz et al., 2001; Zheng et al., 2010). For example, leaf size has been shown to decrease in areas with long‐term grazing (Díaz et al., 2001). Floral and pollinator traits have been less studied than vegetative traits but are necessary to know which interactions can occur and which functional traits are important for particular pollination network structures. For instance, flower shape and rewards can determine interactions with pollinators (Koski et al., 2015; Lázaro et al., 2020). Pollinator traits can determine dispersal distance, energy requirement, and ability to access a flower (Hall et al., 2019). However, no clear trend has been found to connect different extensive grassland management types to the importance of traits on the role of pollinators and flowers in a pollination network (Albrecht et al., 2018; Bartomeus et al., 2018; Lázaro et al., 2020). Different types of grazing and mowing management can add various species filters, for example, type of biomass removal (Díaz et al., 2001; Tälle et al., 2016), light availability (Aavik et al., 2008), and environmental conditions, that could cause functional traits important for network structure to differ between them.

We focused our study on two historically important types of mowing and grazing management that are known to promote plant diversity, but have not been studied in terms of pollinators and pollination networks, mown wooded meadows and grazed alvar pastures. To investigate the influence of these two extensive grassland management types with similar intensity, we here compare their species diversity, species composition, and network structure. The primary hypothesis is that diversity would be comparable, but composition and network structure would differ between the management types. Additionally, we examine how functional traits influence the roles that species play in pollination networks and expect that the species and functional traits important for species‐level network properties would differ among the two management types.

## 2 MATERIALS AND METHODS

### 2.1 Study system

Our study sites are situated in Western Estonia, near the settlement of Virtsu (58.5812, 23.5439). Estonia has a rich history of extensive land use, but socioeconomic shifts have led to an overall decline in grassland area. Nevertheless, in Western Estonia, there are still multiple grasslands that have been continuously extensively maintained (Kukk & Kull, 1997; Pärtel et al., 1999). We sampled the two most common grassland management types in Western Estonia: the mown wooded meadows and grazed alvar pastures. Management intensity is comparably low between them; wooded meadows are mown once a year (Kukk & Kull, 1997) and alvars typically have 0.2–0.5 livestock units (LSU)/hectar (Helm, 2019). Both types are known to be rich in plant and insect species (wooded meadows: 76 plant species/m^2^, alvars: 63 plant species/m^2^; Kukk & Kull, 1997, Pärtel et al., 1999, van Swaay, 2002, Wilson et al., 2012). Wooded meadows and alvar pastures differ in terms of their traditional management, light conditions, soil depth, vegetation type, among other characteristics (Appendix A1). Due to sparse mature tree cover up to 30%, wooded meadows have more heterogeneous light and moisture conditions (Kukk & Kull, 1997), while the dry calcareous alvar pastures are exposed to direct light as they only have shrub cover up to 30% consisting mostly of juniper (*Juniperus communis* L.) (Pärtel et al., 1999). In terms of vegetation type, wooded meadows have the *Scorzonera humilis*‐*Melampyrum nemorosum* association, while alvar pastures typically contain the *Filipendula vulgaris*‐*Trifolium montanum* association (Pärtel et al., 1999).

### 2.2 Sampling design

We sampled plants, pollinators, and plant−pollinator interactions on three wooded meadows and three alvar pastures (see Appendix A for details and a map of the sites) in early July 2018. Ten 30 × 2 m transects with a minimum distance of 20 m from each other were representatively positioned at each site. At each transect, a botanist identified all flowering plants to species (using Krall et al., 1999) and estimated the cover of flowering individuals over the total transect area. Each of the ten transects per site were subsequently surveyed for flower‐visiting insects for 15 min, not including processing time for caught insects, for a total of 150 minutes of observation per site and 450 minutes of observation per management type. During this period, two surveyors slowly walked back and forth along each transect searching for insects (Lepidoptera, Hymenoptera, or Diptera− henceforth also referred to as pollinators) that were interacting with the reproductive parts of flowers and noted down the interaction. Most Lepidoptera and bumblebees were visually identified in the field, while all other insects were collected and pinned. Among insects, 87.6% of individuals were identified to species level and 88.9% were identified to genus level by an entomological specialist (see Acknowledgments). The remainder, mainly non‐syrphid Diptera, were identified to family and morphospecies level.

### 2.3 Statistical analysis

#### 2.3.1 Diversity and composition of plants and pollinators

Even though we sampled each management type with the same effort, these management types might vary in the number of individuals and interactions observed. Thus, rarefaction curves were used to compare standardized species diversity between the two management types and among sites using sample‐ and coverage‐based rarefaction and extrapolation. Sample‐based rarefaction allows comparisons of species richness at a standardized number of sampling units (Colwell et al., 2012). Coverage‐based rarefaction (Chao & Jost, 2012) allows comparisons of species richness at a standardized level of sample completeness (the proportion that the sampled species comprise of the total individuals in the community). We used the “iNEXT” function in the iNEXT R package (Chao et al., 2014; Hsieh et al., 2019) to generate the rarefaction curves for diversity per sampling unit (i.e., transect) and per sample coverage, first, comparing the two management types (sites within each management type are pooled) and second, comparing all six sites. Abundances were converted to incidences (presence or absence) before incidence‐based methods were applied. Data were interpolated and, in the case of coverage, extrapolated to twice the sample size (Chao & Jost, 2012). Nonoverlapping 95% confidence intervals at the same level of coverage or at the total number of transects indicate significant differences at a level of 5% among the expected species richness estimates regardless of whether they are interpolated or extrapolated (Chao & Jost, 2012; Colwell et al., 2012). Hill‐Shannon diversity (exp(Shannon index)) (Hill, 1973) was used as the diversity measure as it can respond to both high and low abundance values and is, in general, the most appropriate Hill number for analyzing the diversity of entire communities (Roswell et al., 2021).

Observed species richness, asymptotic species richness based on the Chao 2 estimator (Chao, 1987), and percent sampling completeness of plants, pollinators, and unique pollination interactions (Chacoff et al., 2012) were calculated for alvar pastures and wooded meadows to assess sampling completeness. We used a Student's *t*‐test to compare the sampling completeness between alvar pastures and wooded meadows, as the data were normal and the two samples had equal variance.

The plant and pollinator species compositions were compared across management types and sites with constrained ordination, specifically, with canonical correspondence analysis (CCA). Pollinator counts and plant cover data were first log (*x *+ 1) transformed to remove the effect of very rare or very common species (ter Braak & Šmilauer, 2015; Warton, 2005) and all further analyses were carried out with the vegan R package (Oksanen et al., 2019). Canonical correspondence analysis (CCA) was selected over redundancy analysis (RDA) and other ordination techniques because we were interested in species relative abundance, rather than richness, at each management type, for which CCA is well‐suited (ter Braak, 1985; ter Braak & Verdonschot, 1995). The gradient lengths of pollinator (3.326 SD – Axis 1) and plant communities (2.206 SD – Axis 1) (estimated by the “decorana” function) were on the lower end of the range recommended for CCA (3.0 or more SDs) that assumes a unimodal response of community composition to explanatory variables (Šmilauer & Lepš, 2014). However, unimodal ordination (i.e., CCA) has a linear face and is therefore also appropriate to apply to data with short gradients. To test whether management type has an effect on species composition, we coded each site with a dummy variable (1: wooded meadow, 2: alvar pasture) and used this as the matrix of explanatory environmental variables. We ran the analysis using the “cca” function and further used the “anova.cca” function to implement ANOVA‐like permutation tests for the significance of management effects with complete enumeration of all possible permutations, in this case, 719 permutations.

#### 2.3.2 Ecological conditions in management types

We calculated the average Ellenberg values (Dzwonko, 2001; Ellenberg et al., 1992) of plants in wooded meadows and alvar pastures to provide a mechanistic understanding of the differences in plant species composition between management types. We used the Ellenberg light indicator values (range from one, shade tolerant, to nine, light tolerant) and moisture indicator values (range from one, drought tolerant, to twelve, water tolerant) taken from http://statedv.boku.ac.at/zeigerwerte. Missing data were removed (13 out of 72 species) to prepare data for analysis. We tested for significant differences in average Ellenberg values between wooded meadows and alvar pastures with the R package npmv (Burchett et al., 2017) for nonparametric multivariate data. Specifically, we used F‐approximations for ANOVA‐type statistics with 1000 permutations. In the case of significance, we followed up with analyses using a subset algorithm determining which Ellenberg indicator values contributed to the significance.

#### 2.3.3 Plant−pollinator interactions

To visualize the pollination network, we generated a web weighted by the interaction frequency using the “plotweb” function in the bipartite R package (Dormann et al., 2008). Pollinators and plants were grouped by genus for this visualization. All further analyses and indices were calculated on the highest level of taxonomic resolution available for plants and pollinators (i.e., species or morphospecies).

#### 2.3.4 Network‐level indices

Five network‐level indices were analyzed to compare the structure of entire bipartite networks across sites and management types (Blüthgen et al., 2006; Thébault & Fontaine, 2010): nestedness, connectance, specialization, interaction diversity, and interaction evenness. Nestedness quantifies asymmetric specialization, that is, specialists interacting with generalists (Blüthgen, 2010). NODF nestedness (Nestedness metric based on Overlap and Decreasing Fill) is more conservative than other nestedness indices and better at avoiding type I errors (Almeida‐Neto et al., 2008; Almeida‐Neto & Ulrich, 2011); it varies from 0 to 100, with 100 meaning perfect nestedness. Connectance is the proportion of possible links that are realized and therefore ranges from 0 to 100, with higher values indicating higher connectance (Dunne et al., 2002). Specialization (H_2_’) describes the deviation of the network from networks expected to occur by chance and provides a measure of the exclusiveness of species interactions (Blüthgen, 2010). It ranges between 0 (no specialization) and 1 (complete specialization). Interaction diversity uses the Shannon Diversity Index; higher values indicate higher diversity and complexity of interactions (Blüthgen, 2010). Interaction evenness uses the Shannon Equitability Index; lower values indicate high variation in interaction frequency while higher values indicate higher interaction evenness (Blüthgen, 2010).

Like estimates of diversity for plants and pollinators, network indices are influenced by the number and completeness of interactions recorded for each site and management type. Thus, we created rarefaction curves for each site and each management type. For each network‐level index, we randomly sampled interactions without replacement until all interactions in the management type were sampled to build an interaction‐based rarefaction curve. Each interaction‐based rarefaction curve is a mean curve of 100 iterations with 95% quantile‐based confidence intervals. Similarly to other types of rarefaction, nonoverlapping confidence intervals at an equal level of interactions indicate significant differences at a level of 5% among the network indices (Chao & Jost, 2012; Colwell et al., 2012; Neuenkamp et al., 2021). We used the function “boot_networklevel” in the package bootstrapnet (Ştefan & Knight, 2020) in R for these analyses. To further confirm this analysis, we ran permutation tests for the interactions by management type, first, with interactions randomly sampled from the wooded meadow pollination network so that it was the same size as the alvar pasture network and second, with both full networks. The null hypothesis for this permutation test states that two observed networks come from the same population of networks. Therefore, we can assume that any difference between the two network indices is due to chance alone with a significance threshold of 5%. More specifically, we randomly assigned network labels to the interactions and computed the network indices and their differences with 1000 iterations. The two‐sided p‐value of the observed network index difference was calculated as the proportion of sample permutations where the absolute difference was greater than or equal to the observed difference.

In addition, weighted null models were generated for each management type using the “nullmodel” function with the method r2dtable and 1000 permutations from the bipartite R package (Dormann et al., 2009). We calculated *z*‐scores to compare the observed network with the null networks and two‐sided *p*‐values for significance of the network indices.

#### 2.3.5 Species‐level indices

We quantified three species‐level indices to capture the role of a species in a network (Vitt et al., 2020): species strength, partner diversity, and species specialization (Bascompte et al., 2006; Blüthgen et al., 2006; Dormann, 2011). Species strength summarizes the dependencies of each species in the network (Bascompte et al., 2006); high values of species strength are interpreted as the importance of a species from its partners’ perspective. Partner diversity uses Shannon's Diversity Index as a measure of the diversity of interactions; it measures a species’ number and distribution of connections with different partners (Dormann, 2011). High values indicate that interactions are spread evenly among partners while low values indicate that interactions are dominated by a few partners. Species specialization (d’) measures how similar the distribution of connections of a species is to a distribution expected under random plant−pollinator interactions and provides a measure of preferential partner selection (Blüthgen et al., 2006). d’ varies between 0 and 1, where higher values indicate higher specialization compared to random expectations.

An interaction‐based rarefaction curve was generated for species‐level indices with the function “boot_specieslevel,” as described above for network‐level indices (Ştefan & Knight, 2020). To further confirm this analysis, for each management type, we sampled all interactions with replacement 1000 times, constructing a sample distribution for each species index. We then applied Kruskal‐Wallis tests with the sampled index as the response variable and species as the explanatory variable. Further, we used pairwise Wilcoxon rank sum tests with the Benjamini‐Hochberg correction for multiple testing for pair‐wise *p*‐values of differences for the species highlighted in the interaction‐based rarefaction curves as having high index values. Our null hypothesis is that there are no significant differences between species indices within a network. We assume a significance threshold of 5%.

#### 2.3.6 Functional trait analysis

We selected a suite of functional traits known to influence plant−pollinator interactions. For each plant species, we collected information on four plant traits (Appendix B1, B2) from the databases LEDA (Kleyer et al., 2008; accessed at December 4, 2018) and BIOLFLOR (Klotz et al., 2002; accessed at December 4th 2018), and for each insect species, we collected information on two insect traits from relevant taxonomic literature (Appendix B1, B2). Plant traits were related to the reliance/specialization of plants on insect pollination (i.e., the amount of floral reward, flower morphology, the prevalence of insect pollination) and flower accessibility (i.e., seed releasing height as a proxy for flower height). Insect traits were related to foraging range, flower choice, and efficiency in acquiring floral resources (Cariveau et al., 2016; i.e., body length and proboscis length). Insect traits were grouped into qualitative size‐based classes (more details in Appendix B1). Only species with more than two observed interactions were included in the modeling approach to exclude data deficient species.

In order to test the influence of species traits on the structure of plant−pollinator networks, we assessed the relationship between species traits, species‐level network indices (i.e., species strength, partner diversity, and specialization), and management type using linear models (*lm* function of the *stats* R package). We built multivariate linear models with each plant and insect species‐based network index as a response variable, and plant and insect traits as well as their interaction with management type as explanatory variables. Number of samples (i.e., plant or insect species) per model only allowed for two‐way interactions between each trait and management type, but not for higher‐level interactions. To avoid collinearity of explanatory variables, we assessed their correlation with chi‐squared tests, suitable for categorical variables. These tests revealed that insect traits were correlated, but plant traits were not (Appendix B3, B4). Therefore, separate models were built for each insect trait as predictors of insect species‐level network indices, while models of plant species‐level network indices included all plant traits in one model.

If model residuals did not meet requirements of normality and variance homogeneity, we log‐transformed the variables. In all cases, model requirements were met after the transformation (if applied). We used type III ANOVA to check for models with significant interactions. As there were no significant interactions, we assessed model differences using type II ANOVA (see Neuenkamp et al., 2021 for a similar approach) since it has been reported as statistically more powerful in the absence of significant interactions (Langsrud, 2003).

All analyses were carried out using R Version 3.6.1 (R Core Team, 2019).

## 3 RESULTS

### 3.1 Diversity and composition of plants and pollinators

In total, we recorded 72 plant species belonging to 22 families, 159 pollinator species belonging to 35 families, and 333 unique interactions between plant and pollinator species. On average, we recorded 36.7 ± 7.4 standard deviations (*SD*) plant and 48.3 ± 16.5 *SD* pollinator species for wooded meadow sites and 33.0 ± 9.2 *SD* plant and 29.7 ± 9.8 *SD* pollinator species for alvar pasture sites. Alvar pastures and wooded meadows did not differ in their sample completeness (Appendix C1; *t*‐test statistic = −0.0627, *df* = 4, *p *= .953).

**FIGURE 1:**
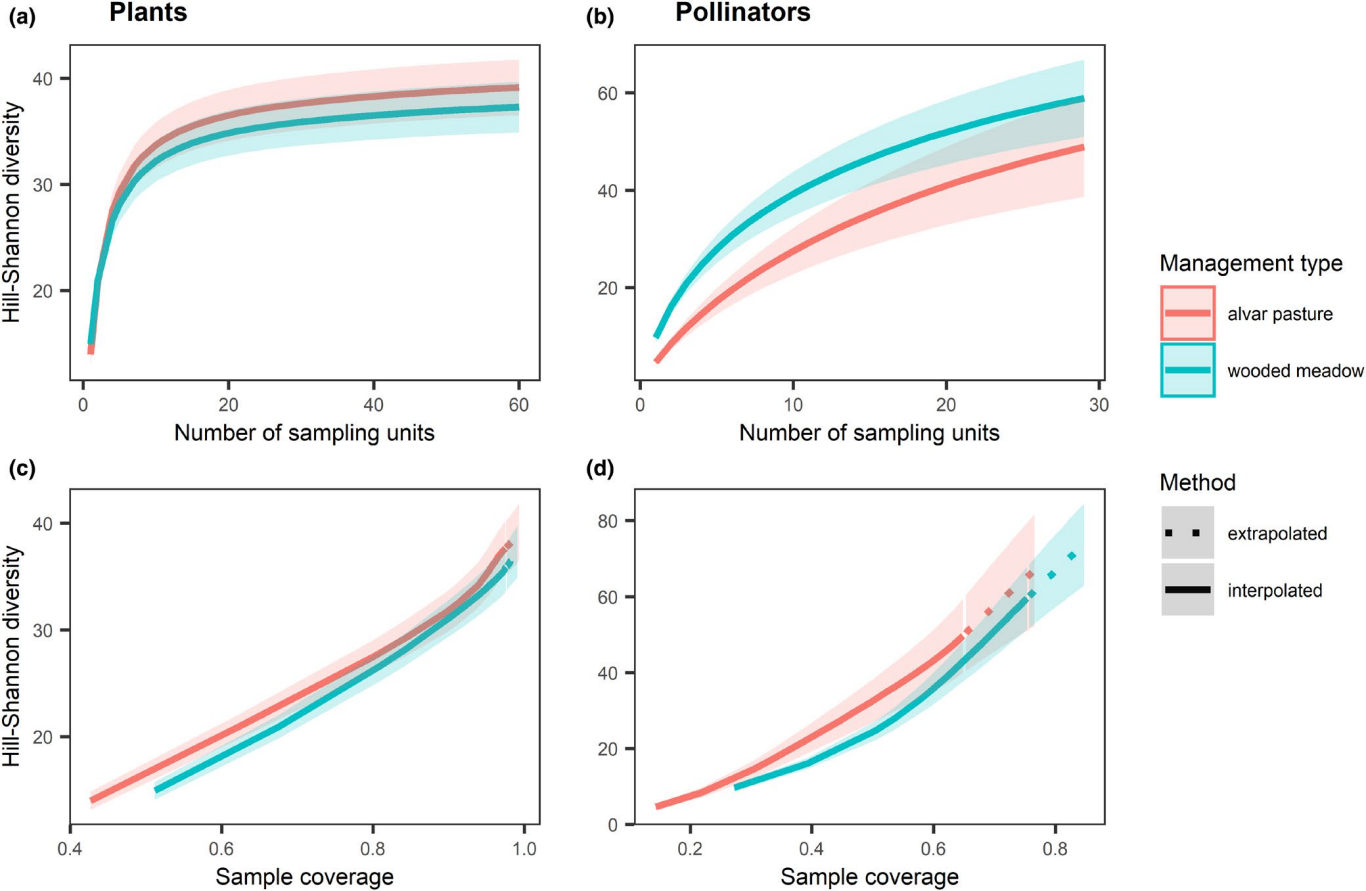
Sample‐based rarefaction curves for (a) plant and (b) pollinator Hill‐Shannon diversity and coverage‐based rarefaction curves for (c) plant and (d) pollinator Hill‐Shannon diversity in wooded meadows (blue) and alvar pastures (red), with 95% confidence intervals. Solid lines are interpolated, while dashed lines are extrapolated

When comparing the species diversity in management types, alvar pastures and wooded meadows were equally diverse for plant (Figure 1a,c) and pollinator species (Figure 1b,d) at equal number of sampling units and sample coverage. When comparing the species diversity at the different sites, plant diversity was only significantly lower for one site, the Viita alvar pasture, for both equal number of sampling units and sample coverage (Appendix C2a,c). However, pollinator diversity in the Viita alvar pasture was higher than the other sites at equal sample coverage (Appendix C2d). When standardizing diversity per sampling unit (i.e., transect), the Allika and Laelatu wooded meadows were significantly more diverse in terms of pollinator species than the Hanila and Laelatu alvar and the Viita wooded meadow (Appendix C2b).

**FIGURE 2:**
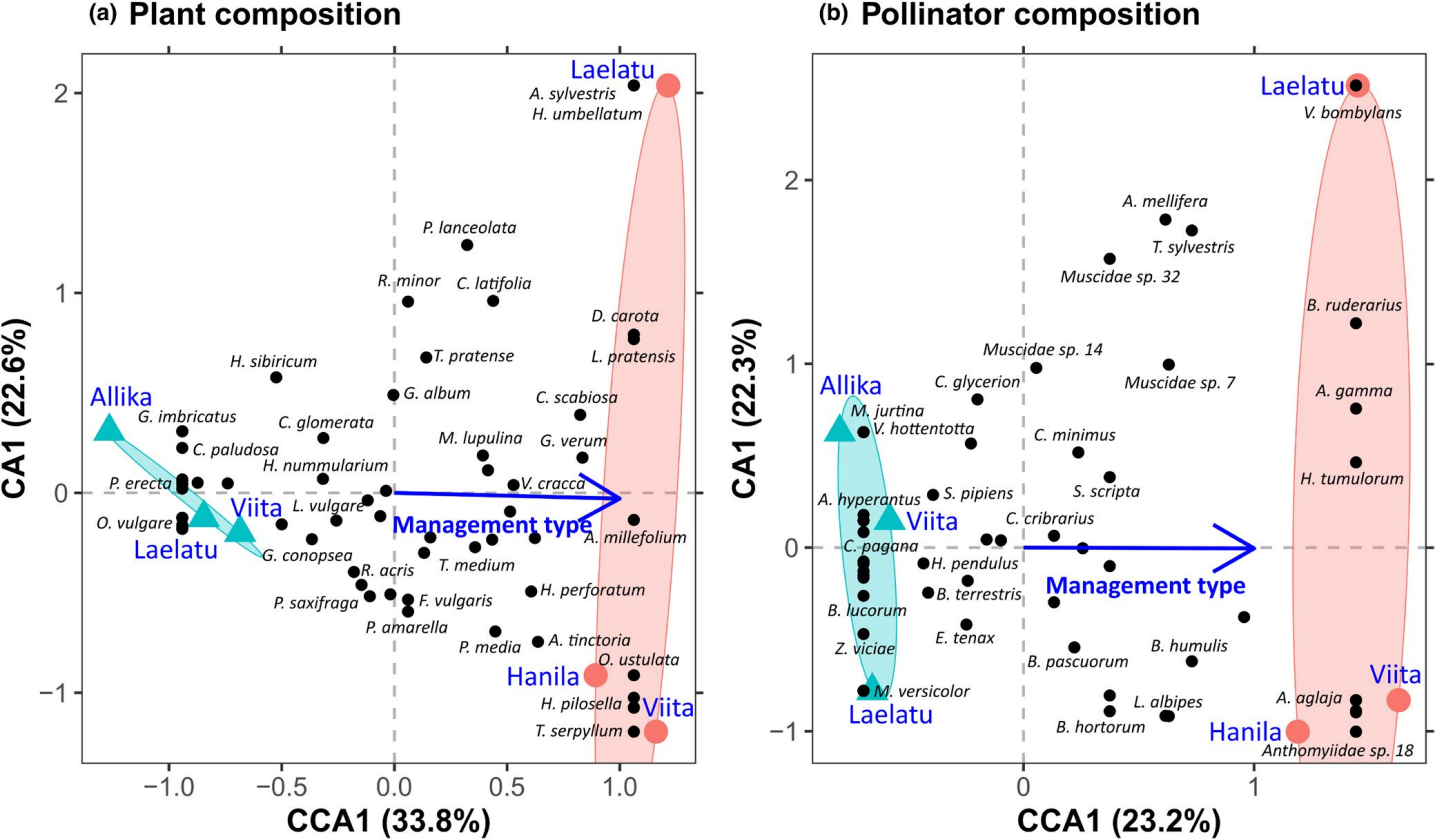
A CCA for (a) plant and (b) pollinator composition, with the first constrained axis (CCA1− management type) charted against the first unconstrained axis (CA1). Species are symbolized by black dots, wooded meadow sites by blue triangles and alvar pasture sites by red dots. Some of the species dots overlap and are labelled representatively, but not exhaustively. The blue arrow represents the direction of management type. Scaling 2, where species are the centroids of the sites and distances between response variable points indicate their χ^2^ distances, was selected for plotting and comparison

The plant and pollinator species composition differed between wooded meadows and alvar pastures (plants: *F* = 2.047, *df* = 1, *p* = .049; pollinators: *F* = 1.212, *df* = 1, *p *= .004). For plants, management type accounted for 33.85% of the variation (Figure 2a), while the first two unconstrained axes of the CCA accounted for 22.58% and 19.89% of the variation, respectively. For pollinators, management type accounted for 23.25% of the variation (Figure 2b), while the first two unconstrained axes accounted for 22.33% and 21.91% of the variation, respectively.

### 3.2 Ecological conditions in management types

Wooded meadows and alvar pastures differed significantly in the average Ellenberg values of their plant species in regards to light and moisture tolerance (nonparametric ANOVA type test, *F* = 58.203, *df*1 = 1.928, *df*2 = 1498.754, *p* < .001). The Ellenberg light and moisture values are significantly different between alvar pastures and wooded meadows (Appendix D), on their own and in combination. Therefore, plant species in wooded meadows were more shade tolerant and water demanding than those in alvar pastures.

### 3.3 Network‐level indices

**FIGURE 3:**
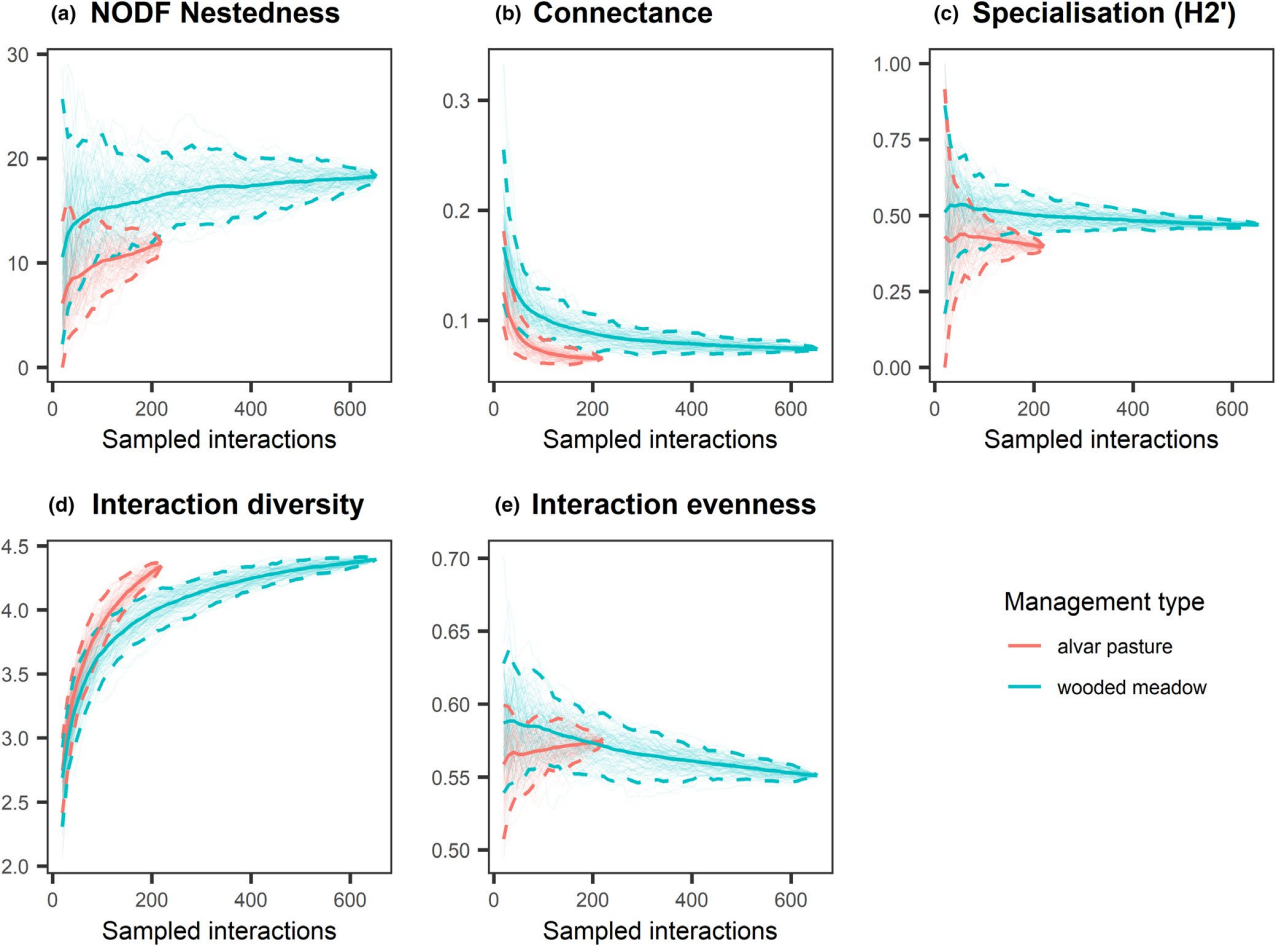
Interaction‐based rarefaction curves of network‐level indices comparing (a) NODF nestedness, (b) connectance, (c) specialisation (H2’), (d) interaction diversity and e) interaction evenness between wooded meadows (blue) and alvar pastures (red). Solid lines and dotted lines indicate mean values and 95% confidence intervals of rarefaction estimates based on 100 iterations, respectively. The endpoint of the curve corresponds to the same value generated by the ‘networklevel’ function in the bipartite R package (Dormann et al., 2009)

At a standardized sampling of interactions, wooded meadows had significantly higher connectance and specialization (H2’), while alvar pastures achieved higher interaction diversity (nonoverlapping 95% quantile‐based confidence intervals and significant *p*‐values, Figure 3b–d, Table 1). Nestedness and interaction evenness did not differ between wooded meadows and alvar pastures (overlapping 95% quantile‐based confidence intervals and nonsignificant *p*‐values, Figure 3a,e, Table 1). According to the null model, all of the indices were significantly different than expected by chance (Appendix E1). When examining sites, the Viita wooded meadow and Hanila alvar had lower interaction diversity than other sites and were less even than the Allika and Laelatu wooded meadows (Appendix E2d,e). The Laelatu and Viita alvar were less specialized than other sites, while nestedness and connectance were indistinguishable among sites (Appendix E2a,b,c).

**TABLE 1:** Results of permutation tests showing differences in network‐level indices per management type, using standardized (equal) sampling and the full networks

	Standardized sampling (*p*‐values)	Full networks (*p*‐values)
NODF nestedness	.12	.05
Connectance	**<.001**	.33
Specialization (H2’)	.**04**	.06
Interaction diversity	.**01**	1.00
Interaction evenness	.85	.09

Note

Values in bold indicate significance (*p* < .05).

### 3.4 Species‐level indices

**FIGURE 4:**
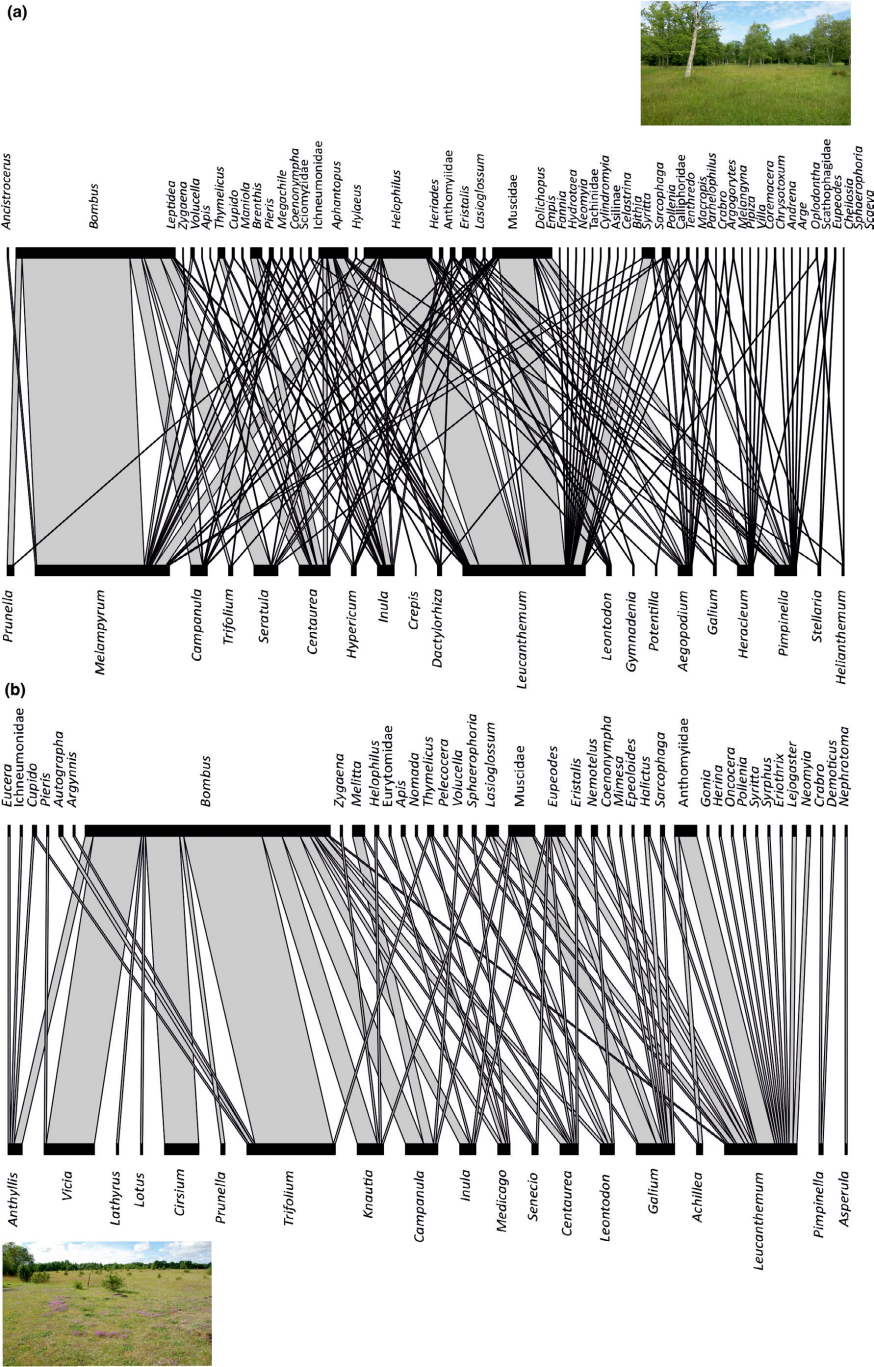
Pollination networks by genus weighted by the interaction frequency in (a) wooded meadows (b) alvar pastures. Photos courtesy of E. Motivans Švara and E. Prangel, respectively

**FIGURE 5:**
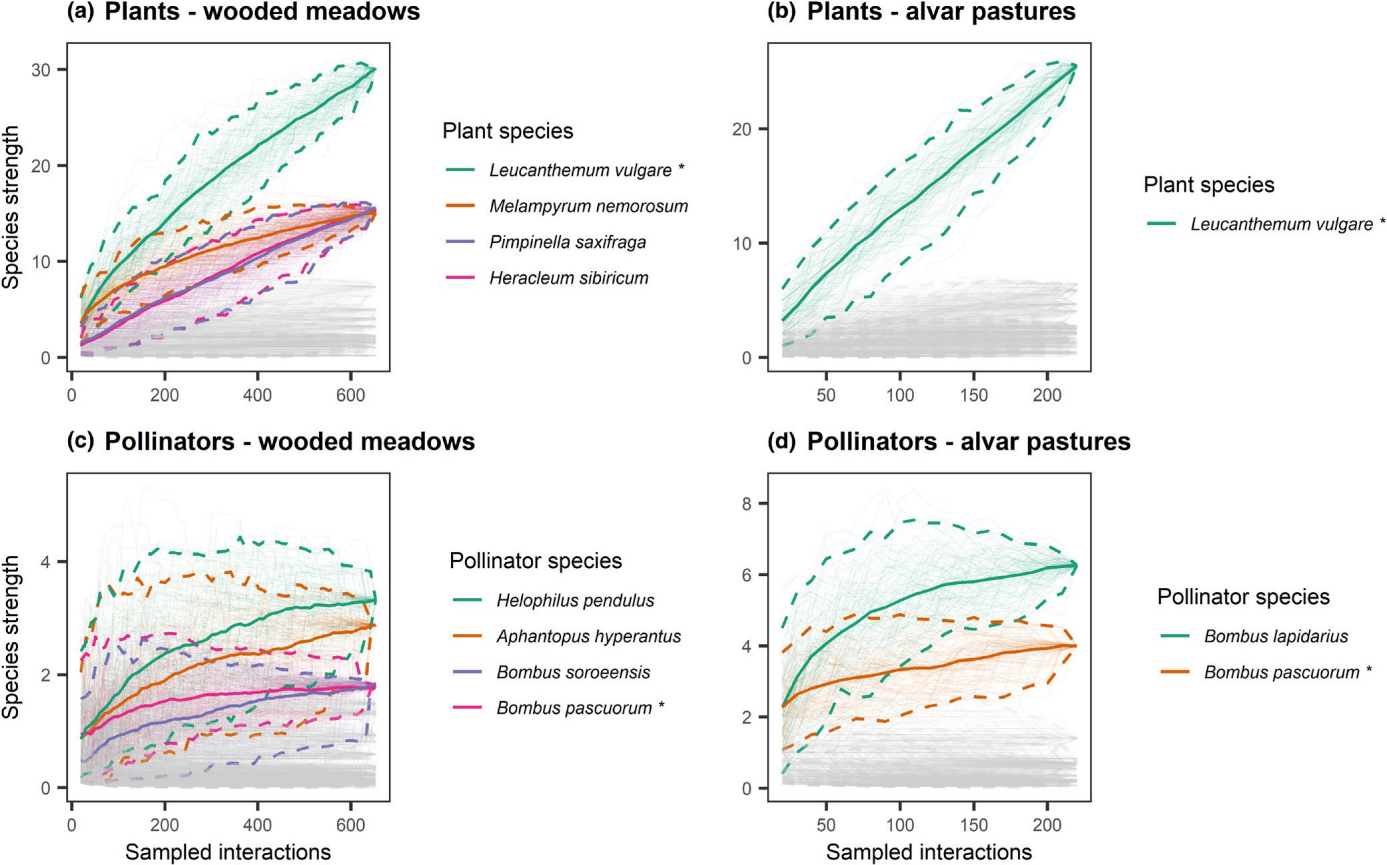
Interaction‐based rarefaction curves of the species‐level network index species strength for plants (a, b), and pollinators (c, d) in wooded meadows (left) and alvar pastures (right). Solid lines indicate mean values and dotted lines indicate 95% confidence intervals of rarefaction estimates based on 100 iterations. The species with the highest species strength (more than half of the maximum species strength) are shown in colour and all other species are grey. An asterisk indicates that the same species appears in both management types, although it may have a different colour. The endpoint of the curve corresponds to the same value generated by the ‘specieslevel’ function in the bipartite R package (Dormann 2011)

Alvar pastures were characterized by 1–2 plant and pollinator species that exhibited strong influence on the pollination network, while more species (3 or more species) shared moderate influence on pollination networks in wooded meadows (Figure 4). For each management type and each species‐level network index sampled, the null hypothesis that there are no significant differences between species indices within a network was rejected (Appendix F1). For alvar pastures, the plant *Leucanthemum vulgare* Lam. and the pollinators *Bombus lapidarius* (L., 1758) and *Bombus pascuorum* (Scop., 1763) had the highest species strength (Figure 5b,d, Appendix F1). For wooded meadows, the plants *L*. *vulgare*, *Melampyrum nemorosum* L., *Pimpinella saxifraga* L., and *Hercleum sibiricum* L. and the pollinators *Helophilus pendulus* (L., 1758) and *Aphantopus hyperantus* (Linnaeus, 1758) had the highest species strength (Figure 5a, c, Appendix F1). Similarly as for species strength, *L*. *vulgare* and *B*. *lapidarius* had significantly higher partner diversity than the other species in alvar pastures (Appendix F1, F2b,d). For wooded meadows, *P*. *saxifraga* and *H*. *sibiricum* had slightly higher partner diversity than other plant species, while *Aphantopus hyperantus* (Linnaeus, 1758) and *Eristalis tenax* (Linnaeus, 1758) had significantly higher partner diversity than other pollinator species (Appendix F1, F2a,c). The plants in both wooded meadows and alvar pastures displayed a comparable range of specialization (Appendix F2e,f). Pollinators in alvar pastures had a higher range of specialization than pollinators in wooded meadows and one bee species, *Melitta haemorrhoidalis* (Fabricius, 1775), was significantly more specialized than the other pollinator species (Appendix F1, F2g,h).

When examining sites, in wooded meadows, *L*. *vulgare* and *M*. *nemorosum* were the plant species with the highest species strength for all sites, and *H*. *pendulus* was the pollinator species with the highest species strength shared for two of the sites, Viita and Laelatu (Appendix F3). In alvar pastures, no plant species with high species strength were found at all sites, while *B*. *lapidarius* was among the species with the highest species strength at all three sites (Appendix F3). Similarly, for partner diversity, *L*. *vulgare* and *M*. *nemorosum* were the plant species with the highest partner diversity for all wooded meadow sites, and the pollinator species *H*. *pendulus* had high partner diversity shared for two of the sites, Viita and Laelatu, but *A*. *hyperantus* also had high partner diversity and was shared by all sites (Appendix F3). In alvar pastures, no plant species with high partner diversity were found at all sites, while *B*. *lapidarius* was among the species with the highest partner diversity at all three sites (Appendix F3). Specialization was site‐dependent for both alvar pastures and wooded meadows, and for plants and insects, though *H*. *sibiricum* was the most specialized plant species for both the Laelatu and Allika wooded meadow sites.

### 3.5 Functional trait analysis

**FIGURE 6:**
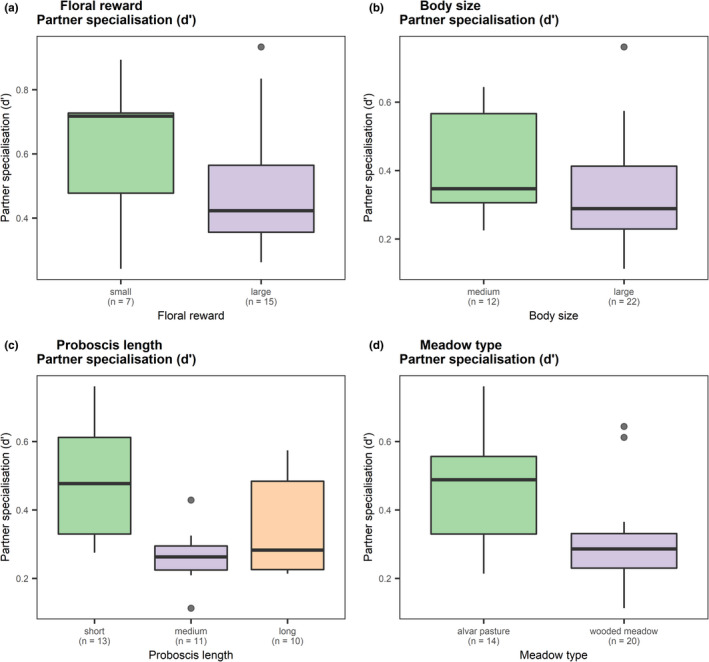
Relationships between species‐level network indices and functional traits for (a) plants and (b–d) pollinators. Box whiskers extend to the 95% confidence intervals around the median. The number of samples (i.e. plant or pollinator species) per group is shown in parentheses below each factor level

Results of linear models show that management type and a subset of three plant and insect functional traits were linked to the species‐level network indices (Tables 2, 3). We did not find significant interaction effects of functional traits and management types (Appendix B5, B6), and thus tested only additive effects of functional traits and management type. For plants, trait and management effects on species‐level network indices were weak with only one marginally nonsignificant (*p *≤ .1) relationship between the trait “amount of pollination reward” and specialization (Table 2). Therefore, plants offering little nectar reward were more specialized to specific pollinators (Figure 6a). For pollinators, both traits and management type were significantly related to specialization, while effects on other species‐level indices were not significant (Table 3). Pollinators with small body size and short proboscis length were more specialized to the flowers of specific plant species (Figure 6b,c). Management type affected partner specialization of pollinators, in such that partner specialization of pollinators was higher in alvar pastures compared to wooded meadows (Figure 6d).

**TABLE 2:** Results of linear models testing the relationship between plant species‐level network indices and plant traits. Estimates are shown for continuous and binary explanatory factors, for the latter they indicate the change from the first to the second factor level as highlighted in brackets after the factor name. The marginally significant (*p *< .1) result is printed in bold and its differences are visualized in Figure 6

Explanatory factors	Response variable											
	log (Species strength)				Partner diversity				Partner specialization (d’)			
	*df*	Estimate	*F*‐value	*p*‐value	*df*	Estimate	*F*‐value	*p*‐value	*df*	Estimate	*F*‐value	*p*‐value
Frequency insect pollination	4		2.189	.127	4		0.989	.448	4		2.391	.104
Amount of floral reward (small – large)	1	0.544	0.770	.396	1	0.087	0.036	.853	1	0.288	4.564	.**052**
Flower morphology (disk – no disk)	1	0.353	0.474	.503	1	−0.017	0.002	.964	1	−0.028	0.063	.806
Max. release height	1	0.958	0.947	.348	1	0.915	1.566	.233	1	0.107	0.253	.624
Management type (alvar pasture – wooded meadow)	1	0.540	1.722	.212	1	0.462	2.283	.155	1	−0.076	0.717	.412

**TABLE 3:** Results of linear models testing the relationship between insect species‐level network indices and insect traits. Estimates are shown for continuous and binary explanatory factors, for the latter they indicate the change from the first to the second factor level as highlighted in brackets after the factor name. Body size class is a three‐level factor (small, medium, large), but the level “small” has been excluded from the analysis, since it was represented by only one pollinator species (see Appendix B1 for details on the assignment of insect traits). Significant (*p *< .05) results are printed in bold and their differences are visualized in Figure 6

Explanatory factors	Response variable											
	log (Species strength)				Partner diversity				Partner specialization (d’)			
	*df*	Estimate	*F*‐value	*p*‐value	*df*	Estimate	*F*‐value	*p*‐value	*df*	Estimate	*F*‐value	*p*‐value
(a) Body size and management type												
Body size class (medium – large)	1	0.765	3.398	.075	1	0.359	4.046	.053	1	−0.103	4.456	.**043**
Management type (alvar pasture – wooded meadow)	1	−0.208	0.267	.609	1	−0.040	0.054	.819	1	−0.163	11.872	.**002**
(a) Proboscis length and management type												
Proboscis length Class	2		2.384	.110	2		3.091	.060	2		6.697	.**004**
Management type (alvar pasture – wooded meadow)	1	−0.471	1.346	.255	1	−0.164	0.897	.351	1	−0.132	9.234	**.005**

Since body size and proboscis length were correlated, we compared the proportion of variance explained by the linear models including these factors. For partner specialization (d’), the linear model (LM) including body size explained substantially more variation than the model including proboscis length (adjusted *R* ^2^(LM_bodysize_) = .28, adjusted *R* ^2^(LM_prob. length_) = .04). Thus, body size might be the more important factor influencing pollinator specialization in the tested meadows. Models including body size or proboscis length explained a similar amount of variation for species strength (adjusted *R* ^2^(LM_bodysize_) = .06, adjusted *R* ^2^(LM_prob. length_) = .07), and partner diversity (adjusted *R* ^2^(LM_bodysize_) = .06, adjusted *R* ^2^(LM_prob. length_) = .09).

## 4 DISCUSSION

Our analyses revealed that two different types of extensive grasslands with comparable management intensity shared many similar features. In support of our primary hypothesis, plant and pollinator diversity were similar, but plant and pollinator composition and network structure differed between the two grassland types. Some aspects of network structure were shared among management types, such as interaction evenness and nestedness, while others, such as connectance, specialization, and interaction diversity, differed. Our second hypothesis that the species and functional traits important for species‐level network structure would differ between the two management types was only partially met as several key species and most functional trait effects were the same for both types, though specialization of individual pollinators was higher in alvar pastures. This shows that although there are unique species and interactions in the management types due to environmental and compositional differences, similar processes are shaping the structure of both pollination networks.

Alvar pastures and wooded meadows contained similar diversity, but different compositions of plants and pollinators, suggesting that both management types are necessary in larger landscapes to support biodiversity. Our results showing high plant diversity under both the mowing and grazing regimes are in line with other studies from this region (Kukk & Kull, 1997; Pärtel et al., 1999; Villoslada Peciña et al., 2019) and with the broader literature on European grasslands (Fontana et al., 2014; Hudewenz et al., 2012; Lázaro, Tscheulin, Devalez, Nakas, & Petanidou, 2016; Lázaro, Tscheulin, Devalez, Nakas, Stefanaki, et al., 2016; Sjödin et al., 2008). While the management types were compositionally different, this was mostly due to differences in the relative abundances of species, corroborating recent findings from the same study area (Villoslada Peciña et al., 2019). The observed differences in plant composition might be due to the lower light availability and higher soil moisture in the wooded meadows. For instance, the mowing management promoted the mass flowering of the partial‐shade‐adapted plant *M*. *nemorosum*, despite mowing usually preventing species’ dominance (Catorci et al., 2011). Grazing may also influence the compositional differences by directly influencing plant species abundance (i.e., through the selective removal of particular plant species; Vázquez & Simberloff, 2003, Yoshihara et al., 2008). The compositional differences of sites within management types were small for wooded meadows, but quite large for alvar pastures. Studies on alvar pasture plant communities in Estonia have shown that there are seven distinct plant composition clusters due to geographic and abiotic differences (Helm, 2019; Pärtel et al., 1999). Although we selected alvar pastures in the same region, it is possible that the distinct Laelatu alvar belongs to a different cluster than the other sites.

Even with overlapping plant and pollinator composition between wooded meadows and alvar pastures, interactions were generally unique to each management type. Despite the uniqueness of interactions, the generalist pollinator *B*. *pascuorum* and plant *L*. *vulgare* played important roles (in terms of species strength and partner diversity) in both wooded meadows and alvar pastures. These findings agreed with Fantinato et al.’s (2019) results that shared pollinator species have higher species strength, but not with their results that shared plant species had lower species strength than species found in a single management type. For alvars, these shared species played disproportionately important roles in maintaining network structure, making the pollination network more vulnerable to the extinction of the key species (Saavedra et al., 2011). For wooded meadows, these species also played important roles but were complemented by other plant and pollinator species, including a butterfly, *A*. *hyperantus*, and a syrphid, *H*. *pendulus*, playing moderate roles. Given these species’ morphological differences from bumblebees in terms of size and proboscis length, it suggests that complementary floral resources were available in high enough quantities to support high abundance of these different species (Fornoff et al., 2017). Correspondingly, the other plant species in wooded meadows playing moderate roles encompassed different flower shapes including lip flowers and disk flowers with open nectar. The pollinator species other than bumblebees in alvar pastures played important roles with their specialization, that is, in visiting flowers that were otherwise rarely visited (Blüthgen, 2010). The species that contributed to the high species‐level specialization in alvar pastures were site‐dependent and not universal for all alvars; site dependency of species‐level specialization has also found in other habitat types (Koski et al., 2015). Therefore, bumblebees and *L*. *vulgare* were important for the pollination networks of both wooded meadows and alvar pastures, but were complemented by other species with high species strength and partner diversity in wooded meadows, and by other species with high specialization in alvar pastures.

The greater specialization of individual species in alvar pastures can be explained by their functional traits. Plants with small amounts of nectar were more specialized to specific pollinators and pollinators with small body sizes and short proboscis lengths were more specialized in the flowers that they visited, with a stronger effect in alvar pastures. The amount of nectar reward is correlated to flower size so flowers with a smaller nectar reward are generally smaller (Harder & Cruzan, 1990). As pollinators size‐match with the flowers they visit (Klumpers et al., 2019; Stang et al., 2009), smaller pollinators visit these flowers. Small flowers are a stronger filter for pollinator size than large flowers, as large flowers can still be visited by small pollinators, but the reverse is not true, which explains the higher specialization, though flower size has been both positively (Koski et al., 2015) and negatively (Lázaro et al., 2020) correlated to specialization. The management type may have also played a role as sheep selectively graze herbs (Dumont et al., 2011; Fraser et al., 2009; Sebastià et al., 2008), reducing their abundance and often reducing the diversity of pollinator groups (Scohier et al., 2013). Therefore, there were few abundant pollinators across the alvar pastures, meaning that most species, besides bumblebees, were rare and thus contributed to high species‐level specialization (Blüthgen, 2010).

Management and species abundance played a strong role in shaping pollination network structure. In contrast to research that found that extensive mowing produces networks with a smaller size and higher connectance than pastures (Kovács‐Hostyánszki et al., 2019), we found larger networks and higher connectance in the wooded meadows. This could be due to wooded meadows being special types of hay meadows with more heterogeneous soil, nutrient, and light conditions due to the sparse tree layer, thus containing high diversity (Aavik et al., 2008). These environmental conditions may promote the growth of the mass‐flowering plant *M*. *nemorosum*, a partial‐shade‐adapted plant, which could have additional effects on network structure, such as specialization. Wooded meadows had higher specialization at the network level, despite higher specialization at the species level in alvar pastures, possibly due to the domination of the network by bumblebees and *M*. *nemorosum*. *M*. *nemorosum* is a member of the Lamiaceae family, which has hidden nectar rewards that are only available to specific pollinators, namely bumblebees (Mänd et al., 2002), rather than a generalist plant species, which could contribute to this higher specialization at the network level. Interaction diversity is higher in the alvar pastures, likely due to wooded meadow interactions being more dominated by *L*. *vulgare* and *M*. *nemorosum* across all sites and containing more redundant interactions. The similar nestedness could be attributed to the abundance in both management types of short‐tongued, generalist bumblebees (Goulson et al., 2005), which interacted with many plant species that were otherwise not frequently visited. These interactions shaped the networks of both management types and therefore also influenced the comparable evenness. All in all, we also see the influence of a few common interactions (Vázquez & Simberloff, 2003), namely between *M*. *nemorosum* and *Bombus* sp., contributing to the differences between the alvar pastures and wooded meadows.

Our results highlight the importance of interaction‐based rarefaction on network‐ and species‐level indices (Ştefan & Knight, 2020; Terry, 2019). Despite comparable sampling effort in the two management types, we observed fewer pollinator individuals and thus fewer interactions in the alvar pastures compared to the wooded meadows. Comparing indices for a standardized number of interactions gives a different result than if we would have compared the indices for each management type using all observed interactions. For example, the wooded meadows and alvar pasture network‐level indices were not different from each other when comparing their full networks. As the abundance of interactions observed per equal sampling effort might vary across many types of ecological factors of interest, this analysis can be used for future plant−pollinator studies.

Our understanding of plant−pollinator interactions in traditionally managed alvar pastures and wooded meadows could be expanded upon in future research. Our data collection includes a short temporal and spatial sampling grain, and thus represents a snapshot of the diversity, composition, and interactions at both management types. It could therefore be expanded across larger spatial (e.g., including all seven clusters of alvar pastures known across the broader region) and temporal grains (e.g., including seasonal and annual variation). In addition, although we studied plant visitation networks, we recognize that not all insects visiting the flowers may play an active role in pollen transfer. In general, butterflies and small flies are not considered to be efficient pollen transporters due to their morphology (Barrios et al., 2016; Larson et al., 2001; Orford et al., 2015), while bumblebees, honeybees, large flies, and wild bees transport pollen more efficiently (Jauker et al., 2012; Rader et al., 2009). In addition, insects may be performing actions other than pollination, such as observed nectar robbing of *M*. *nemorosum* by short‐tongued bumblebees.

All in all, the coexistence of both species‐rich habitats helps to boost regional species and interaction diversity (Fantinato et al., 2019). We found that there was some species turnover between wooded meadows and alvar pastures and that each hosted unique interactions that nonetheless resulted in similarly nested and even network structure (Koski et al., 2015). There were key connector pollinator species in the *Bombus* genus that helped to contribute to the stability of the networks and are known to pollinate crops in the surrounding landscape (Marja et al., 2018). Unsurprisingly, these two management types have the highest proportion of ecosystem service hotpots in Estonia, but unfortunately only make up 10% of the country's seminatural grassland cover (Villoslada Peciña et al., 2019). Therefore, it is important to protect these habitats in the face of grassland decline. The European Union‐funded LIFE to alvars project (https://life.envir.ee/english‐project‐life‐alvars) in Estonia helped to restore alvar habitats and enable their regular management. However, wooded meadows need more attention; they are declining at a particularly alarming rate throughout Europe, as they are labor intensive to maintain and difficult to scale up to more intense management (Centeri et al., 2016). This study shows that they, as well as alvar pastures, are valuable conservation areas due to their plant, pollinator, and interaction diversity.

## 5 CONCLUSIONS

Our results demonstrate that extensive management via mowing (wooded meadows) and grazing (alvar pastures) in seminatural grasslands creates conditions that support diverse plant and pollinator communities, which enable the development of even and nested pollination networks. The same generalist plant and pollinator species were important for the pollination networks of both wooded meadows and alvar pastures. They were complemented by other species with high species strength and partner diversity in wooded meadows, and by other species with high specialization in the alvar pastures. Extensively managed, biodiverse grasslands can therefore serve as important source populations of plant and pollinator diversity and provide stable pollination networks. Conservation and restoration of these habitats counteract the general decline of plant and insect diversity in the surrounding, mostly intensively managed, European agricultural landscape (Seibold et al., 2019). However, the low‐intensity agricultural practices needed to maintain biodiverse seminatural grasslands are becoming increasingly rare due to socioeconomical changes (Ceballos et al., 2010; Strijker, 2005). Therefore, it is important to preserve different types of high‐value natural grasslands to increase the regional species pool, support different interactions, and maintain pollination services to the surrounding landscape.

## CONFLICT OF INTEREST

The authors declare that they have no conflict of interest.

## AUTHOR CONTRIBUTION

**Elena Motivans Švara:** Conceptualization (equal); Data curation (equal); Formal analysis (equal); Investigation (equal); Methodology (equal); Project administration (lead); Visualization (lead); Writing‐original draft (equal); Writing‐review & editing (lead). **Valentin Ştefan:** Data curation (equal); Formal analysis (equal); Methodology (equal); Software (lead); Visualization (equal). **Esther Sossai:** Data curation (equal); Investigation (equal); Writing‐review & editing (supporting). **Reinart Feldmann:** Data curation (supporting); Investigation (equal); Project administration (lead); Writing‐review & editing (supporting). **Dianne Joy Aguilon:** Investigation (equal); Writing‐review & editing (supporting). **Anna Bontsutsnaja:** Investigation (equal); Writing‐review & editing (supporting). **Anna E‐Vojtkó:** Investigation (equal); Writing‐review & editing (supporting). **Isabel C. Kilian:** Investigation (equal); Writing‐review & editing (supporting). **Piret Lang:** Investigation (equal); Writing‐review & editing (supporting). **Marilin Mõtlep:** Investigation (equal); Writing‐review & editing (supporting). **Elisabeth Prangel:** Investigation (equal); Writing‐review & editing (supporting). **Mari‐Liis Viljur:** Investigation (equal); Writing‐review & editing (supporting). **Tiffany M. Knight:** Conceptualization (equal); Funding acquisition (lead); Investigation (lead); Project administration (lead); Resources (lead); Supervision (equal); Writing‐original draft (equal); Writing‐review & editing (equal). **Lena Neuenkamp:** Conceptualization (equal); Formal analysis (lead); Supervision (lead); Writing‐original draft (equal); Writing‐review & editing (equal).

## Supporting information

Supplementary Material

## Data Availability

The results of the plant−pollinator interaction survey and flowering plant survey, as well as Appendix B2 and F1 are available on Dryad. Doi: https://doi.org/10.5061/dryad.gqnk98snn.
