# Supplementary material for: Effects of different types of low‐intensity management on plant‐pollinator interactions in Estonian grasslands
Source: Ecol Evol. 2021 Nov 22;11(23):16909–26. doi: 10.1002/ece3.8325 (PMC8668793; doi:10.1002/ece3.8325)
Supplement: Supplementary file 1 — Supplementary Material [file ECE3-11-16909-s001.docx]

**Supporting Information ̶ Effects of different types of low-intensity management on plant-pollinator interactions in Estonian grasslands, Motivans Švara et al.**

Appendix A: Site information.

A1. Summary of environmental parameters and other relevant characteristics for each study site.

| Management type | Site | Latitude/  Longitude (decimal degrees) | Management history | Mean number of flowering plant species per transect | Mean flower cover per transect (%) | Mean tree cover per transect (%) |
| --- | --- | --- | --- | --- | --- | --- |
| Alvar pastures | Hanila | 58.6287, 23.5317 | Grazed in the past, now mown; seedbank containing typical alvar composition | 18.7 | 16.2 | 0 |
|  | Laelatu | 58.5801, 23.5580 | Continuously grazed (< 100 years) | 13.1 | 13.6 | 0 |
|  | Viita | 58.7770, 23.7933 | Continuously grazed (< 100 years) | 10.6 | 7.7 | 0 |
| Wooded meadows | Allika | 58.7169, 23.7718 | Continuously mown; 30-50% woody cover; ground vegetation height 30-40cm | 12.7 | 13.9 | 14.3 |
|  | Laelatu | 58.5844, 23.5696 | Continuously mown since late 18^th^ century; 30-50% woody cover; ground vegetation height 30-40cm | 16.7 | 21.1 | 14.3 |
|  | Viita | 58.7711, 23.7904 | Continuously mown; 50-70% woody cover; ground vegetation height 40-50cm | 15.5 | 16.1 | 10.5 |

Additional environmental data*,** - Hanila alvar pasture: pH^b^ (KCL)=7, N^b^ (%)=0.4, P^b^ (mg/kg)=20.0, K^b^ (mg/kg)= 120.0, orgC^b^ (%)=4.6, Allika wooded meadow: T(C°)Jan^a^=-2.5, T(C°) Jul^a^= 17.8, Mean ann. precip._mm_^a^=626, Laelatu wooded meadow: pH^b^ (KCL)=7, N^b^ (%)=0.6, P^b^ (mg/kg)=23.4, K^b^ (mg/kg)= 107.1, orgC^b^ (%)=11.4.

* average values 1980-2010; http://www.ilmateenistus.ee**soil data for alvar pastures exemplarily for the Hanila alvar pasture (as documented by Neuenkamp et al. 2018); soil data for wooded meadows exemplarily for the Laelatu wooded meadow (as documented by Aavik et al. 2008)

A2. A map showing the locations of the sampled sites.


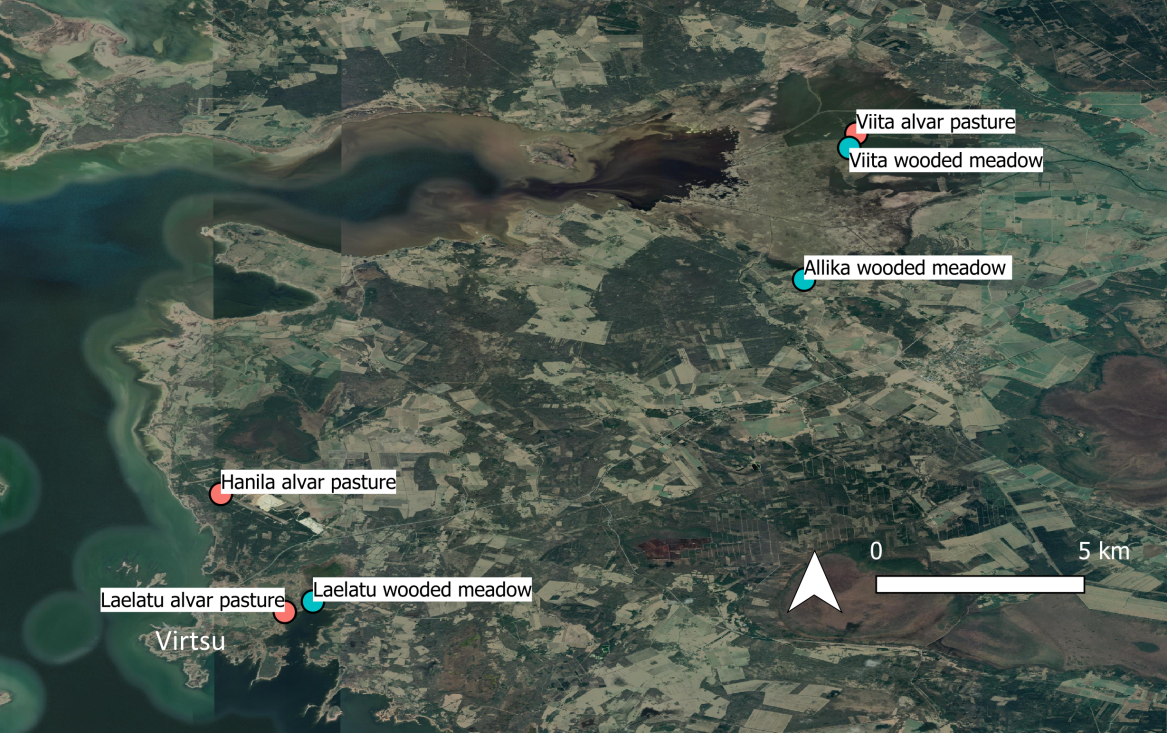


Appendix B: Information related to functional traits and models.

B1. Description of selected plant and insect functional traits.

| Level | Trait | Measurement | Ecological meaning | Source |
| --- | --- | --- | --- | --- |
| Plant | Maximum (seed) release height | Plant height from the ground to the highest flower, used as a proxy for maximum plant height (Kleyer et al. 2008: Leda Trait Standards 6.1.). Trait measures were available for all 29 species. | Maximum seed release height together with seed terminal velocity is classically used as indicator for plant seed dispersal by wind, but also by animals (high release height = good dispersal probability). Estimates on the position of the highest flower can additionally inform about the accessibility of flowers as sources of nectar for insects (high, exposed flowers =high attraction of pollinating insects). | Kleyer et al. 2008 |
|  | Flower morphology | Flower types according to Kugler (1970); manual aggregation of the different flower classes to disk flowers and non-disk flowers. Disk flowers = types 1 (‘Scheibenblume’) + 7 (‘Körbchenblume’); non-disk flowers = other. Categories were evaluated based on the visual flower appearance for species where no type was assigned resulting in trait information for all 29 species. | Flower morphology can determine how easily pollinators can land on the flower and access the nectar. Disk flowers offer a large surface for pollinators to land on and search for nectar, vice versa for non-disk flowers. Disk flowers typically belong to plants that have a generalist pollination system and are accessible by a wide range of different pollinator species. | Klotz, S., Kühn, I., & Durka 2002 |
| Plant | Floral reward | Amount of floral reward (mostly nectar, but also pollen, oil or deception) provided by flowers that attracts insects. Data on the BIOLFLOR database (Klotz, S., Kühn, I., & Durka 2002) was presented in five classes (none, little, present, plenty, unknown); we divided the data into two classes: high (plenty) and low (none, little, present) reward. “Unknown” reward was coded as NA. For five species with missing values with extremely small flowers, floral reward was set to “small”. This led to traits for 24 of 29 species. | Complementary to the flower accessibility, floral reward can indicate the attractiveness of a flower to an insect as food or other resource source. In the case of nectar reward, it might indicate the probability of a pollinator obtaining nectar when visiting the flower as they are more likely to have a steady nectar supply. | Klotz, S., Kühn, I., & Durka 2002 |
| Plant | Prevalence of insect pollination | Descriptor of how frequently a plant species is pollinated by insects ranging in five categories from 'always' to 'never'. Category 'always' indicates strong dependency on insect pollination while 'never' indicates the opposite. Trait measures were available for 24 of 29 species. | Plant species can influence their attractiveness to pollinating insects by making their pollination an easy task and rewarding in terms of nectar supply, through changes in plant morphology and nectar production. The investments in these adjustments, however, are likely influenced by the dependence of plants on insect pollination as reflected by the frequency of insect pollination. | Klotz, S., Kühn, I., & Durka 2002 |
| Insect | Proboscis length | The length of the proboscis, grouped into size classes, Short: 0-3.99 mm, Medium: 4-7.99 mm, Long >8 mm. Trait measures were available for 31 of 41 species. | Proboscis length influences the flower choice of pollinators (Harder 1985) and their foraging behaviour (Harder 1983). The longer the tongue, the greater the flower depth from which the pollinator can still access nectar and the larger the range of flowers the pollinator can use for foraging. | Herrera 1989; **Hymenoptera:** Goulson et al. 2005, Fortel et al. 2014, Cariveau et al. 2016; **Lepidoptera:** Jennersten 1983, Paulus, H.F., Krenn 1996, Okamoto et al. 2008, Sexton 2014, Krenn and Bauder 2018; **Diptera:** Kabos 1964, 1975, Gilbert 1985, Tschorsnig and Herting 1994, Erzinçlioğlu 1996, Barták et al. 2016 |
| Insect | Body size | Approximate whole body length, grouped into size classes, Small: 0-4.99mm, Medium: 5-9.99mm, Large >10mm. Diptera (non-Syrphid) were measured in the laboratory. Trait measures were available for 35 of 41 species. | Body size is also correlated to foraging range (Greenleaf et al. 2007), but mostly indicates nectar demand. | Kühsel and Blüthgen 2015; **Hymenoptera:** Loken 1973, Witt 1998, Amiet et al. 1999, 2004, 2007, Mauss et al. 2004, **Syrphidae:** Dusek and Laska 1976, van Veen 2009, Klecka J. Hadrava J., Biella P. 2018. |

Appendix B2. Functional traits of all insect species, including species name, family name, proboscis length (mm), proboscis size class (short, medium, long), proboscis literature reference, body length (mm), body length size class (small, medium, large), and body length literature reference. Excel file uploaded in Dryad, DOI: https://doi.org/10.5061/dryad.gqnk98snn.

Appendix B3. Collinearity between predictors of species-level indices of plant-pollinator networks for pollinator species, shown by p-values of chi-squared tests (suitable for categorical factors or the combination of continuous and categorical factors). P-values < 0.05 indicate significant collinearity. For collinear predictors, we calculated separate linear models. Predictors were a) management type, a two-level factor (alvar pasture, wooded meadow), b) body size class, a three-level factor (small, medium, and large body size), and c) proboscis length class, a three-level factor (short, medium and, long proboscis length). Species were assigned to trait classes based on a literature review (see Appendix B1 for details on the methods and Appendix B2 for the specific references). Since only one insect species with a short body size was found, this class was omitted from the analysis, leading to a body size class factor with only two levels (medium, large).

|  | Management type | Body size class |
| --- | --- | --- |
| Management type | 1.000 |  |
| Body size class | 0.748 | 1.000 |
| Proboscis length class | 0.514 | **<0.001** |

Appendix B4. Collinearity between predictors of species indices of plant-pollinator networks for plant species, shown by p-values of chi-squared tests (suitable for categorical factors or combination of continuous and categorical factors). P-values < 0.05 indicate significant collinearity. Predictors were: a) management type, a two-level factor (alvar pasture, wooded meadow), b) frequency of insect pollination, a five-level factor (always, common, sometimes, seldom, exceptional), c) floral reward, a two-level factor (low, high), d) flower morphology, a two-level factor (disk flower, no disk flower) and e) maximal release height, a continuous factor (unit cm). Species were assigned to trait classes or values based on information in the Leda and BIOLFLOR trait database (see Appendix B1 for details).

|  | Management Type | Frequency insect pollination | Floral reward | Flower morphology |
| --- | --- | --- | --- | --- |
| Management type | 1.000 |  |  |  |
| Frequency insect pollination | 0.735 | 1.000 |  |  |
| Floral reward | 0.770 | 0.092 | 1.000 |  |
| Flower morphology | 1.000 | 0.479 | 0.921 | 1.000 |
| Release height | 0.735 | 0.286 | 0.217 | 0.426 |

Appendix B5: Results of Type III ANOVA assessing the effect of predictor interactions for species-level network indices of pollinators a) species strength, b) partner diversity, and c) specialisation (d’), separately for models of body size and proboscis length class. Separate models were used for the two predictors due to their collinearity (Appendix B3). No significant interactions were found, and thus were removed from the final model. The final model was then tested using Type II ANOVA, since it can be statistically more powerful than Type III ANOVA in the absence of interactions of explanatory factors. See Table 3 in the main text for the final model results.

| **a) Response: Species strength** |  |  |  |
| --- | --- | --- | --- |
| Factor | DF | F-Value | P-value |
| **1. Model: log (Species strength) --> Body size class*Management type** | | | |
| Body size class | 1 | 0.524 | 0.475 |
| Management type | 1 | 0.019 | 0.892 |
| Body size class*Management | 1 | 0.241 | 0.627 |
| Residuals | 30 |  |  |
| **2. Model: log(Species strength) --> Proboscis length class*Management type** | | | |
| Proboscis length class | 2 | 0.456 | 0.638 |
| Management type | 1 | 0.374 | 0.546 |
| Proboscis length class*Management | 2 | 0.108 | 0.898 |
| Residuals | 28 |  |  |
|  |  |  |  |
| **b) Response: Partner diversity** |  |  |  |
| Factor | DF | F-Value | P-value |
| **1. Model: Partner diversity --> Body size class*Management type** | | | |
| Body size class | 1 | 0.874 | 0.357 |
| Management type | 1 | 0.000 | 0.990 |
| Body size class*Management | 1 | 0.122 | 0.729 |
| Residuals | 30 |  |  |
| **2. Model: Partner diversity --> Proboscis length class*Management type** | | | |
| Proboscis length class | 2 | 2.006 | 0.153 |
| Management type | 1 | 0.134 | 0.717 |
| Proboscis length class*Management | 2 | 0.607 | 0.552 |
| Residuals | 28 |  |  |
|  |  |  |  |
| **c) Response: Partner specialisation (d’)** |  |  |  |
| Factor | DF | F-Value | P-value |
| **1. Model: Partner specialisation --> Body size class*Management type** | | | |
| Body size class | 1 | 0.692 | 0.412 |
| Management type | 1 | 9.692 | 0.004 |
| Body size class*Management | 1 | 0.313 | 0.580 |
| Residuals | 30 |  |  |
| **2. Model: Partner specialisation --> Proboscis length class*Management type** | | | |
| Proboscis length class | 2 | 3.056 | 0.063 |
| Management type | 1 | 3.146 | 0.087 |
| Proboscis length class*Management | 2 | 0.087 | 0.917 |
| Residuals | 28 |  |  |

Appendix B6. Results of Type III ANOVA assessing the effect of predictor interactions for species network indices of plant species: a) species strength, b) partner diversity, and c) specialisation (d’). All predictors were tested in the same model due to the missing evidence of collinearity (Appendix B4). Interactions were restricted to two-level interactions due to insufficient sample sizes for three-way interactions. No significant interactions were found, and thus were removed from the final model. The final model was then tested using Type II ANOVA, since it can be statistically more powerful than Type III ANOVA in the absence of interactions of explanatory factors. See Table 2 in the main text for the final model results.

| **a) Response: Species strength** |  |  |  |
| --- | --- | --- | --- |
| **Model: log(Species strength) --> Frequency insect pollination*Management type + Floral reward*Management type + Flower morphology*Management type + Max. release height*Management type** | | | |
| Factor | DF | F-Value | P-value |
| Frequency insect pollination | 4 | 1.033 | 0.462 |
| Management type | 1 | 1.606 | 0.252 |
| Floral reward | 1 | 0.176 | 0.690 |
| Flower morphology | 1 | 0.002 | 0.962 |
| Max. release height | 1 | 0.154 | 0.708 |
| Freq. ins. pol.*Management | 4 | 2.231 | 0.182 |
| Fl. reward*Management | 1 | 2.437 | 0.170 |
| Fl. morph.*Management | 1 | 0.074 | 0.794 |
| Rel. height*Management | 1 | 4.618 | 0.075 |
| Residuals | 6 |  |  |
|  |  |  |  |
| **b) Response: Partner diversity** |  |  |  |
| **Model: Partner diversity --> Frequency insect pollination*Management type + Floral reward*Management type + Flower morphology*Management type + Max. release height*Management type** | | | |
| Factor | DF | F-Value | P-value |
| Frequency insect pollination | 4 | 0.380 | 0.816 |
| Management type | 1 | 1.096 | 0.335 |
| Floral reward | 1 | 0.046 | 0.838 |
| Flower morphology | 1 | 0.187 | 0.680 |
| Max. release height | 1 | 0.013 | 0.913 |
| Freq. ins. pol.*Management | 4 | 0.826 | 0.554 |
| Fl. reward*Management | 1 | 1.220 | 0.312 |
| Fl. morph.*Management | 1 | 0.000 | 0.991 |
| Rel. height*Management | 1 | 2.043 | 0.203 |
| Residuals | 6 |  |  |
|  |  |  |  |
| **c) Response: Partner specialisation (d’)** |  |  |  |
| **Model: Partner specialisation --> Frequency insect pollination*Management type + Floral reward*Management type + Flower morphology*Management type + Max. release height*Management type** | | | |
| Factor | DF | F-Value | P-value |
| Frequency insect pollination | 4 | 1.714 | 0.264 |
| Management type | 1 | 0.097 | 0.766 |
| Floral reward | 1 | 2.471 | 0.167 |
| Flower morphology | 1 | 0.257 | 0.630 |
| Max. release height | 1 | 0.371 | 0.565 |
| Freq. ins. pol.*Management | 4 | 0.529 | 0.720 |
| Fl. reward*Management | 1 | 0.697 | 0.436 |
| Fl. morph.*Management | 1 | 0.171 | 0.693 |
| Rel. height*Management | 1 | 0.694 | 0.437 |
| Residuals | 6 |  |  |

Appendix C. Sampling completeness and rarefaction of plant and pollinator species.

C1. Observed richness, asymptotic richness, and percent sampling completeness of plants, pollinators, and unique plant-pollinator interactions for wooded meadows and alvar pastures.

|  |  | Wooded meadow | Alvar pasture |
| --- | --- | --- | --- |
| Plant species | Observed richness | 56.00 | 54.00 |
|  | Asymptotic richness | 61.68 | 64.08 |
|  | Percent sampling completeness | 90.79 | 84.27 |
| Pollinator species | Observed richness | 112.00 | 74.00 |
|  | Asymptotic richness | 357.45 | 186.76 |
|  | Percent sampling completeness | 31.33 | 39.96 |
| Unique plant-pollinator interactions | Observed richness | 215.00 | 126.00 |
|  | Asymptotic richness | 1017.22 | 926.00 |
|  | Percent sampling completeness | 21.14 | 13.61 |

**
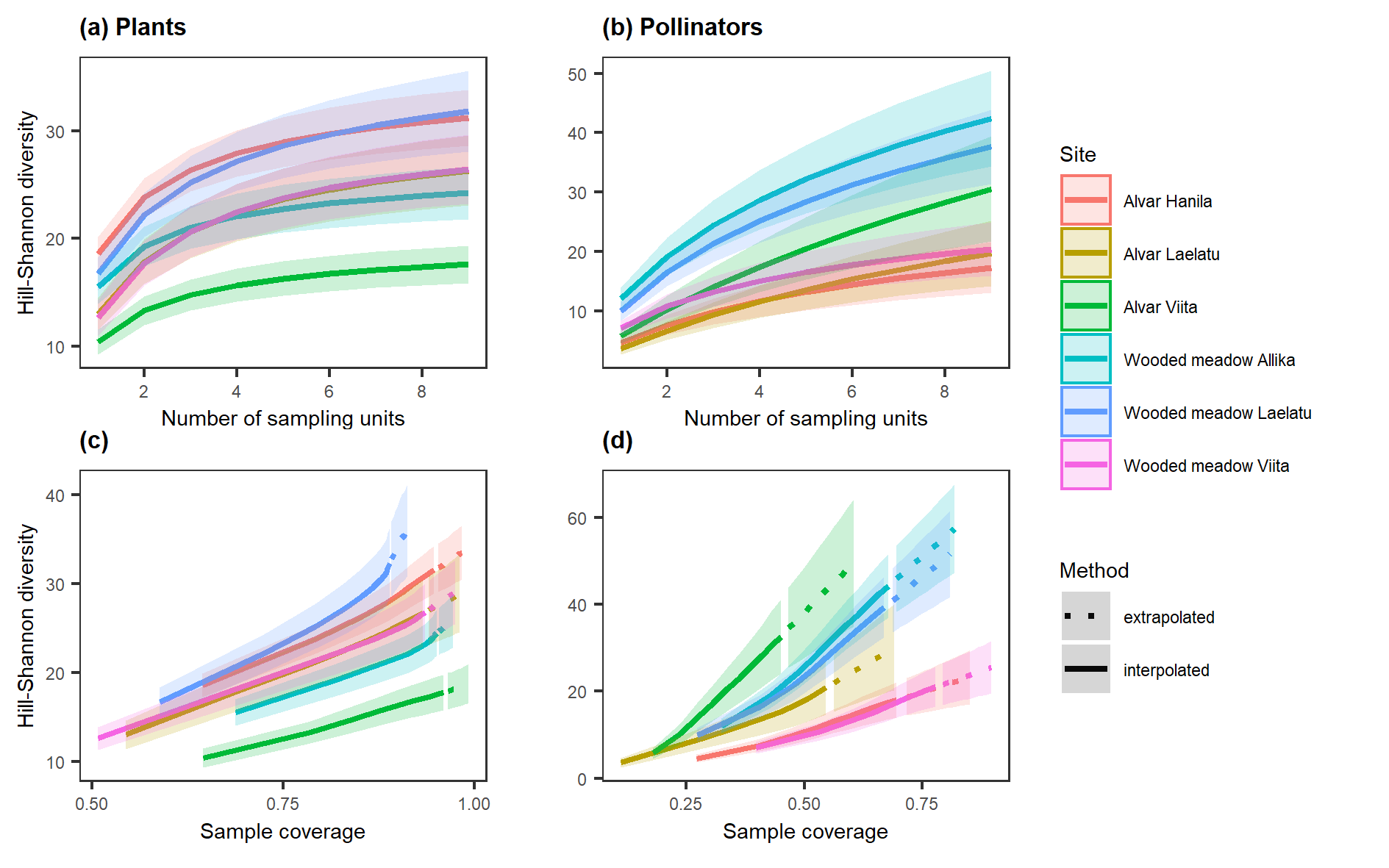
**

C2. Sampling unit-based rarefaction curves for a) plant and b) pollinator Hill-Shannon diversity and coverage-based rarefaction curves for c) plant and d) pollinator Hill-Shannon diversity for each site, with 95% confidence intervals. Solid lines are interpolated, while dashed lines are extrapolated.

Appendix D: Ellenberg values per management type.


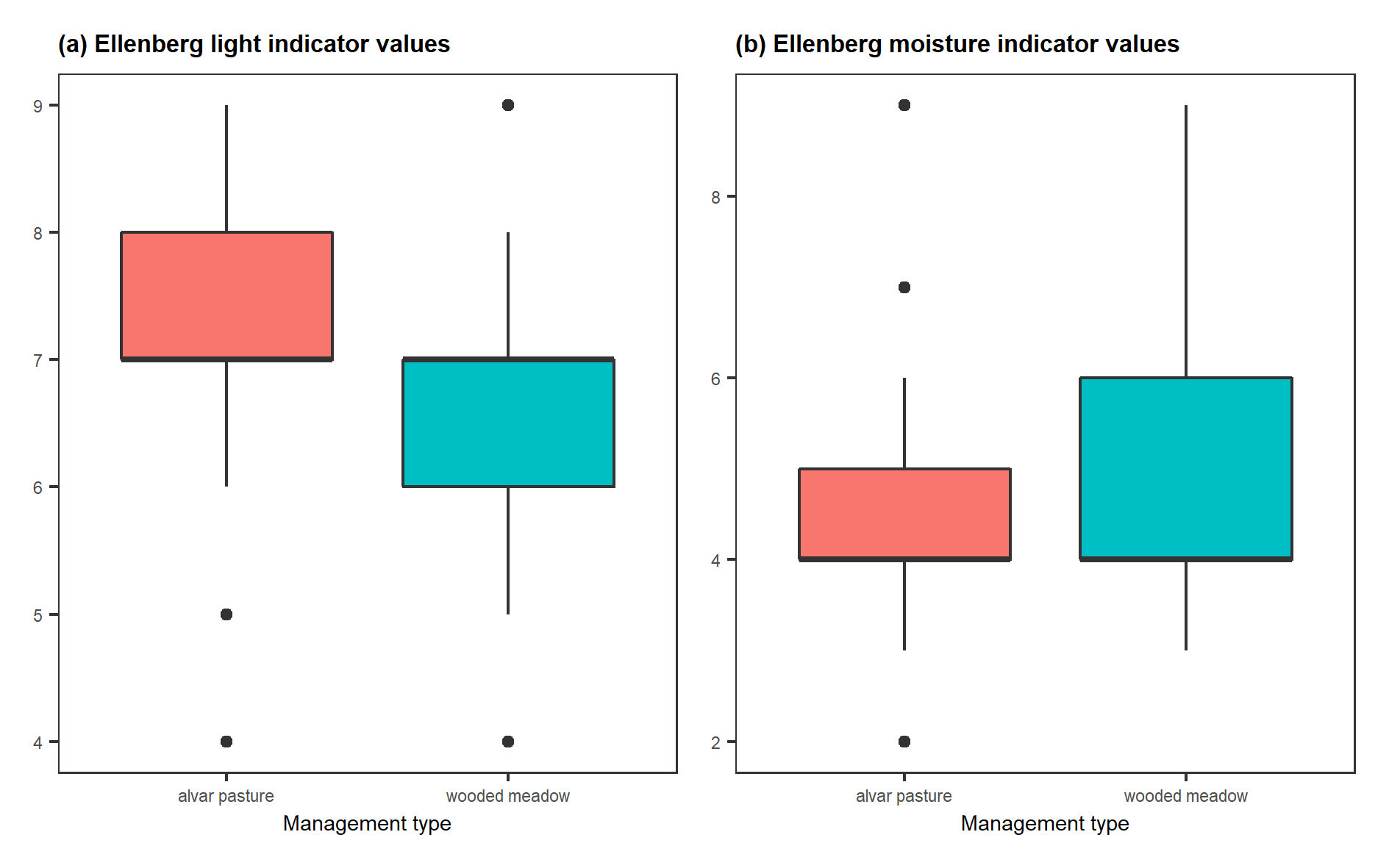


D1. The average Ellenberg a) light and b) moisture indicator values for the plant species found in alvar pastures and wooded meadows.

Appendix E: Network-level indices.

E1. P-values of the null models ran to discriminate whether the network indices were significantly different than those occurring by chance.

|  | Wooded meadow | | Alvar pasture | |
| --- | --- | --- | --- | --- |
|  | z-score | p-value | z-score | p-value |
| NODF | -6.528 | <0.001 | -2.029 | <0.05 |
| H2 | 14.673 | <0.001 | 3.364 | <0.001 |
| Interaction evenness | -14.673 | <0.001 | -3.364 | <0.001 |
| Interaction diversity | -14.673 | <0.001 | -3.364 | <0.001 |
| Connectance | -8.167 | <0.001 | -4.285 | <0.001 |
|  |  |  |  |  |

**
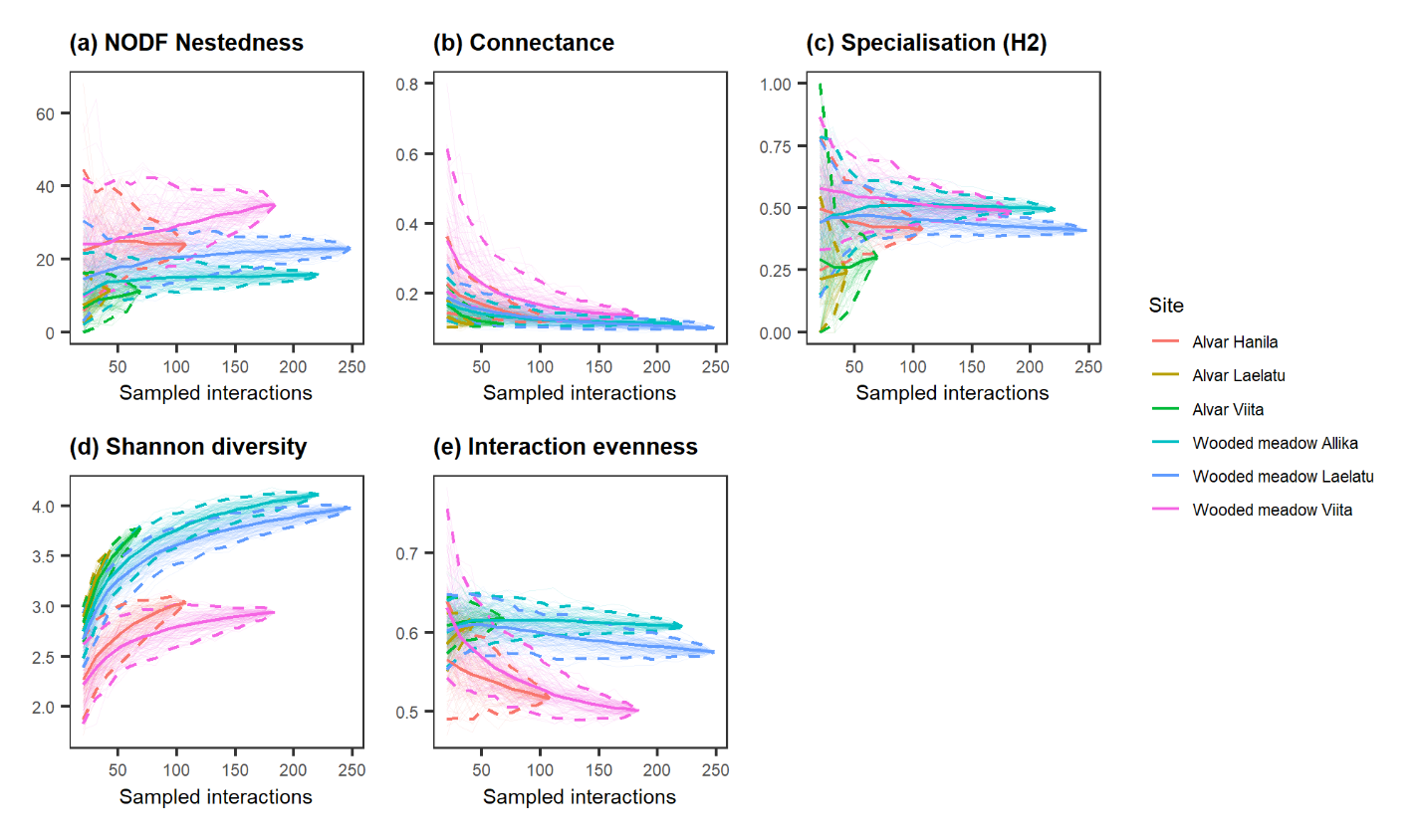
**E2. Interaction-based rarefaction curve of network-level indices comparing a) nestedness, b) connectance, c) specialisation (H2), d) interaction diversity and e) interaction evenness among sites. Solid lines and dotted lines indicate mean values and 95% confidence intervals of rarefaction estimates based on 1000 iterations, respectively.

Appendix F: Species-level indices.

F1. Results of Kruskal-Wallis tests testing for significant differences between species indices (species strength, partner diversity, specialisation d’) of the species within each management type and results of Wilcoxon rank sum tests with the Benjamini-Hochberg correction for multiple testing for pair-wise p-values of differences between species (results are shown for the species highlighted in colour in Figure 5 and Appendix F2). CSV file uploaded in Dryad, DOI: https://doi.org/10.5061/dryad.gqnk98snn.


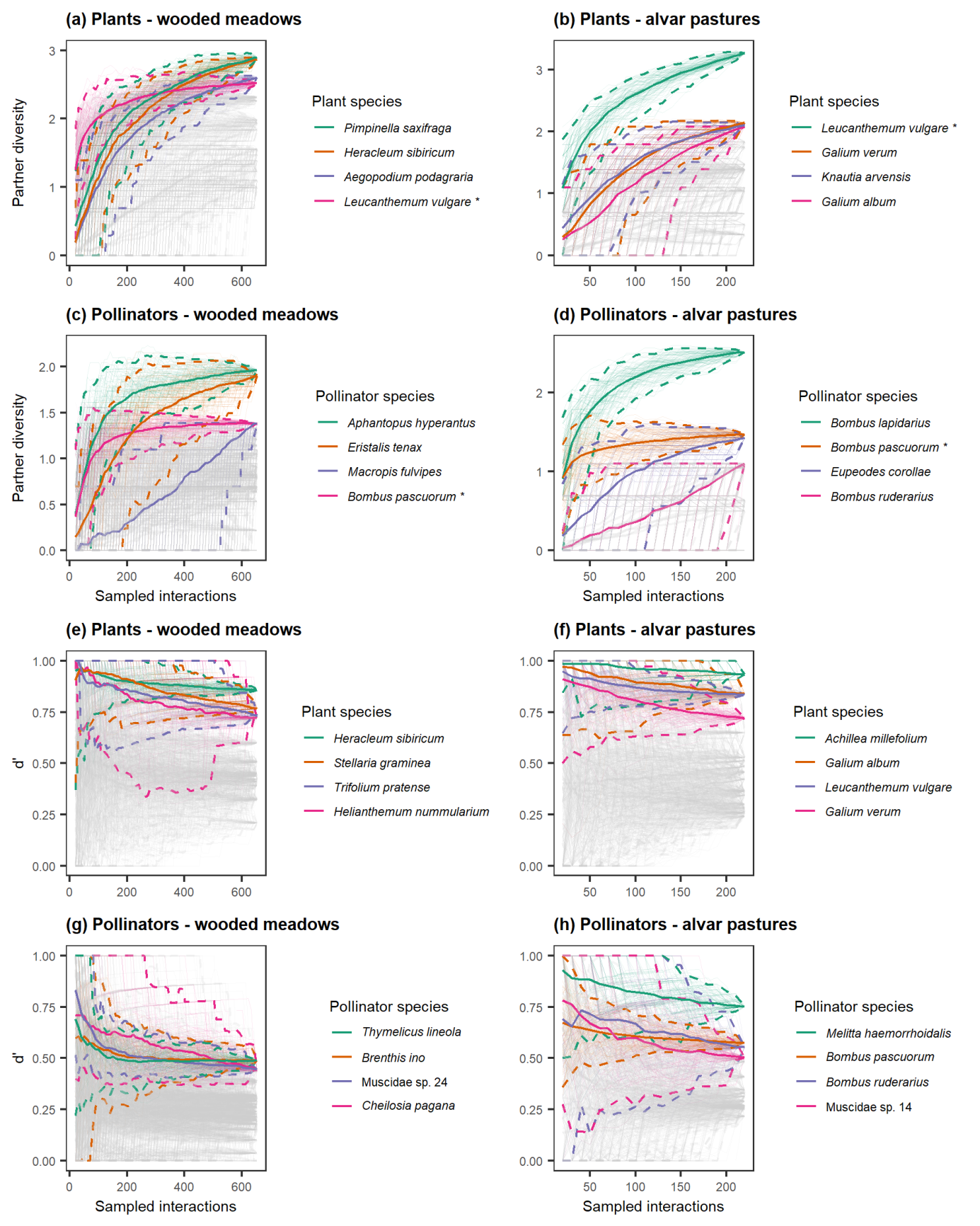


F2. Interaction-based rarefaction curve for partner diversity (a, b: plants, c, d: pollinators) and specialisation d’ (e, f: plants, g, h: pollinators), with wooded meadows on the left and alvar pastures on the right. Solid lines indicate mean values and dotted lines indicate 95% confidence intervals of rarefaction estimates based on 100 iterations. The first four species are shown in colour and all other species are grey. An asterisk indicates that the same species appears in both management types, although it may have a different colour.

F3. The species-level network indices, species strength, partner diversity, and specialization (d’), for the four species with the highest values at each site.

|  | Index | Plant species with highest index value | Value | Pollinator species with highest index value | Value |
| --- | --- | --- | --- | --- | --- |
| Hanila alvar | Species strength | *Trifolium medium* | 4.804 | *Bombus pascuorum* | 5.476 |
|  |  | *Knautia arvensis* | 4.335 | *Bombus lapidarius* | 2.724 |
|  |  | *Leontodon hispidus* | 3.584 | *Eupeodes corollae* | 1.125 |
|  |  | *Inula salicina* | 2.784 | *Nephrotoma* sp. | 1.000 |
|  |  |  |  | *Eupeodes nielseni* | 1.000 |
| Viita alvar | Species strength | *Leucanthemum vulgare* | 26.667 | *Bombus lapidarius* | 3.455 |
|  |  | *Centaurea scabiosa* | 2.667 | *Bombus ruderarius* | 1.000 |
|  |  | *Cirsium acaule* | 2.333 | *Argynnis aglaja* | 1.000 |
|  |  | *Pimpinella saxifraga* | 2.000 | *Eupeodes corollae* | 0.698 |
| Laelatu alvar | Species strength | *Galium album* | 7.333 | *Bombus lapidarius* | 2.650 |
|  |  | *Galium verum* | 5.167 | *Melitta haemorrhoidalis* | 2.000 |
|  |  | *Anthyllis vulneraria* | 2.000 | *Volucella bombylans* | 1.250 |
|  |  | *Trifolium pratense* | 1.958 | *Bombus ruderarius* | 1.250 |
| Laelatu wooded meadow | Species strength | *Pimpinella saxifraga* | 18.089 | *Helophilus pendulus* | 3.012 |
|  |  | *Leucanthemum vulgare* | 10.755 | *Eristalis tenax* | 2.303 |
|  |  | *Melampyrum nemorosum* | 7.071 | *Bombus lapidarius* | 2.115 |
|  |  | *Seratula tinctoria* | 5.106 | *Bombus pascuorum* | 1.583 |
| Viita wooded meadow | Species strength | *Melampyrum nemorosum* | 12.156 | *Helophilus pendulus* | 3.071 |
|  |  | *Leucanthemum vulgare* | 10.968 | Muscidae sp. 1 | 2.198 |
|  |  | *Hypericum maculatum* | 2.333 | *Bombus lapidarius* | 1.319 |
|  |  | *Leontodon hispidus* | 2.198 | *Syritta pipiens* | 1.020 |
| Allika wooded meadow | Species strength | *Leucanthemum vulgare* | 16.409 | *Aphantopus hyperantus* | 3.130 |
|  |  | *Heracleum sibiricum* | 15.854 | *Bombus soroeensis* | 1.30 |
|  |  | *Melampyrum nemorosum* | 8.242 | *Bombus pascuorum* | 1.117 |
|  |  | *Aegopodium podagraria* | 5.277 | *Bombus terrestris* | 0.920 |
| Hanila alvar | Specialisation (d’) | *Senecio jacobaea* | 1.000 | *Nephrotoma* sp. | 1.000 |
|  |  | *Asperula tinctoria* | 1.000 | *Eupeodes nielseni* | 1.000 |
|  |  | *Achillea millefolium* | 1.000 | Anthomyiidae sp. 4 | 0.808 |
|  |  | *Galium verum* | 0.829 | Anthomyiidae sp. 18 | 0.808 |
| Viita alvar | Specialisation (d’) | *Trifolium medium* | 1.000 | *Bombus ruderarius* | 1.000 |
|  |  | *Prunella vulgaris* | 1.000 | *Argynnis aglaja* | 1.000 |
|  |  | *Pimpinella saxifraga* | 1.000 | *Melitta leporina* | 0.805 |
|  |  | *Leucanthemum vulgare* | 0.775 | *Demoticus* sp. | 0.805 |
|  |  |  |  | *Crabro cribrarius* | 0.805 |
| Laelatu alvar | Specialisation (d’) | *Medicago lupulina* | 1.000 | *Sphaerophoria scripta* | 1.000 |
|  |  | *Leucanthemum vulgare* | 1.000 | Anthomyiidae sp. 5 | 1.000 |
|  |  | *Anthyllis vulneraria* | 1.000 | *Melitta haemorrhoidalis* | 0.753 |
|  |  | *Galium album* | 0.873 | Ichneumonidae sp. | 0.667 |
|  |  |  |  | *Cupido minimus* | 0.667 |
| Laelatu wooded meadow | Specialisation (d’) | *Heracleum sibiricum* | 1.000 | *Sarcophaga* sp. 5 | 1.000 |
|  |  | *Galium verum* | 1.000 | *Parhelophilus versicolor* | 1.000 |
|  |  | *Galium album* | 1.000 | *Melangyna umbellatarum* | 1.000 |
|  |  | *Pimpinella saxifraga* | 0.756 | *Scaeva selenitica* | 0.758 |
| Viita wooded meadow | Specialisation (d’) | *Trifolium pratense* | 0.905 | *Pieris rapae* | 0.849 |
|  |  | *Hypericum maculatum* | 0.894 | *Lasioglossum villosulum* | 0.760 |
|  |  | *Aegopodium podagraria* | 0.736 | Anthomyiidae sp. 2 | 0.760 |
|  |  | *Leucanthemum vulgare* | 0.541 | Muscidae sp. 9 | 0.649 |
|  |  |  |  | Anthomyiidae sp. 15 | 0.649 |
| Allika wooded meadow | Specialisation (d’) | *Heracleum sibiricum* | 0.834 | *Hylaeus confusus* | 0.682 |
|  |  | *Stellaria graminea* | 0.774 | *Sphaerophoria scripta* | 0.660 |
|  |  | *Leucanthemum vulgare* | 0.606 | Calliphoridae sp. 2 | 0.605 |
|  |  | *Melampyrum nemorosum* | 0.594 | Anthomyiidae sp. 17 | 0.605 |
| Hanila alvar | Partner diversity | *Knautia arvensis* | 1.979 | *Bombus lapidarius* | 1.807 |
|  |  | *Leontodon hispidus* | 1.748 | *Bombus pascuorum* | 1.391 |
|  |  | *Inula salicina* | 1.386 | *Bombus pratorum* | 1.055 |
|  |  | *Trifolium medium* | 1.296 | *Lasioglossum albipes* | 0.693 |
|  |  |  |  | *Eupeodes corollae* | 0.693 |
|  |  |  |  | *Bombus soroeensis* | 0.693 |
| Viita alvar | Partner diversity | *Leucanthemum vulgare* | 3.224 | *Bombus lapidarius* | 1.490 |
|  |  | *Centaurea scabiosa* | 1.386 | *Eupeodes corollae* | 0.868 |
|  |  | *Cirsium acaule* | 1.149 | *Halictus tumulorum* | 0.693 |
|  |  | *Galium verum* | 1.099 | *Eristalis tenax* | 0.693 |
|  |  |  |  | *Bombus soroeensis* | 0.693 |
|  |  |  |  | *Bombus pascuorum* | 0.693 |
| Laelatu alvar | Partner diversity | *Galium album* | 2.079 | *Bombus lapidarius* | 1.494 |
|  |  | *Galium verum* | 1.946 | *Thymelicus sylvestris* | 1.099 |
|  |  | *Trifolium pratense* | 1.386 | Muscidae sp. 14 | 1.099 |
|  |  | *Medicago sativa* | 1.055 | *Melitta haemorrhoidalis* | 1.055 |
| Laelatu wooded meadow | Partner diversity | *Pimpinella saxifraga* | 2.900 | *Eristalis tenax* | 1.987 |
|  |  | *Leucanthemum vulgare* | 2.442 | *Helophilus pendulus* | 1.927 |
|  |  | *Melampyrum nemorosum* | 1.989 | *Bombus lapidarius* | 1.476 |
|  |  | *Seratula tinctoria* | 1.980 | *Aphantopus hyperantus* | 1.386 |
| Viita wooded meadow | Partner diversity | *Melampyrum nemorosum* | 2.175 | *Coenonympha glycerion* | 1.040 |
|  |  | *Leucanthemum vulgare* | 1.757 | *Aphantopus hyperantus* | 1.011 |
|  |  | *Leontodon hispidus* | 1.332 | *Helophilus pendulus* | 0.789 |
|  |  | *Hypericum maculatum* | 1.099 | Ichneumonidae sp. | 0.693 |
|  |  |  |  | *Cupido minimus* | 0.693 |
|  |  |  |  | *Bombus sylvarum* | 0.693 |
| Allika wooded meadow | Partner diversity | *Heracleum sibiricum* | 2.811 | *Aphantopus hyperantus* | 1.852 |
|  |  | *Leucanthemum vulgare* | 2.367 | *Macropis fulvipes* | 1.386 |
|  |  | *Aegopodium podagraria* | 2.098 | *Bombus terrestis* | 1.215 |
|  |  | *Melampyrum nemorosum* | 1.950 | *Pieris napi* | 1.099 |

References:

1. Amiet, F., M&#x000FC;ller, A., & Neumeyer, R. et al (1999). Apidae 2: Colletes, Dufourea, Hylaeus, Nomia, Nomioides, Rhophitoides, Rophites, Sphecodes, Systropha. Fauna Helvetica, 4(1-219). Neuch&#x000E2;tel: Schweizerische Entomologische Gesellschaft.
2. Amiet, F., Herrmann, M., M&#x000FC;ller, A., & Neumeyer, R. et al (2004). Apidae 4: Anthidium, Chelostoma, Coelioxys, Dioxys, Heriades, Lithurgus, Megachile, Osmia, Stelis. Fauna Helvetica, 91-273). Neuch&#x000E2;tel: Schweizerische Entomologische Gesellschaft.
3. Amiet, F., Herrmann, M., M&#x000FC;ller, A., &#x00026; Neumeyer, R.</givenNames></author> et al (2007). Apidae 5: Ammobates, Ammobatoides, Anthophora, Biastes, Ceratina, Dasypoda, Epeoloides, Epeolus, Eucera, Macropis, Melecta, Melitta, Nomada, Pasites, Tetralonia, Thyreus, Xylocopa. .Fauna Helvetica</bookSeriesTitle>, 20(1-356). Neuch&#x000E2;tel: Schweizerische Entomologische Gesellschaft.
4. Bart&#x000E1;k, M., Preisler, J., Kub&#x000ED;k, &#x00160;., &#x00160;ul&#x000E1;kov&#x000E1;, H., &#x00026; Sloup, V. (2016). Fanniidae (Diptera): new synonym, new records and an updated key to males of European species of Fannia. ZooKeys, 91-115.
5. Dusek, J.</givenNames></author> &#x00026; Laska, P. (1976). European species of Metasyrphus: key, descriptions and notes (Diptera, Syrphidae). Acta entomologica bohemoslovaca, 73, 263&#x02010;282.
6. Erzin&#x000E7;lio&#x0011F;lu, Z. (1996). Blowflies. Richmond Publishing Co. Ltd.
7. Fortel, L., Henry, M., Guilbaud, L., Guirao, A. L., Kuhlmann, M., Mouret, H., Rollin, O., &#x00026; Vaissi&#x000E8;re, B. E. et al (2014). Decreasing abundance, increasing diversity and changing structure of the wild bee community (Hymenoptera: Anthophila) along an urbanization gradient. PLoS One, 9, e104679.
8. Gilbert, F. S. (1985). Morphometric patterns in hoverflies (Diptera, Syrphidae). Proceedings of the Royal Society B: Biological Sciences, 224, 79-90. <https://doi.org/10.1098/rspb.1985.0022>
9. Greenleaf, S. S., Williams, N. M., Winfree, R., &#x00026; Kremen, C. (2007). Bee foraging ranges and their relationship to body size. Oecologia, 153, 589-596. https://doi.org/10.1007/s00442&#x02010;007&#x02010;0752&#x02010;9
10. Harder, L. D. (1985). Morphology as a predictor of flower choice by bumble bees. Ecology, 66, 198-210. <https://doi.org/10.2307/1941320>
11. Harder, L. D. (1983). Flower handling efficiency of bumble bees: morphological aspects of probing time. Oecologia, 57, 274-280. <https://doi.org/10.1007/BF00379591>
12. Herrera, C. M. (1989). Pollinator abundance, morphology, and flower visitation rate: analysis of the &#x00022;quantity&#x00022; component in a plant&#x02010;pollinator system. Oecologia, 80, 241-248.
13. Jennersten, O. (1983). Butterfly Visitors as Vectors of Ustilago violacea Spores between Caryophyllaceous Plants. Oikos, 40, 125-130. <https://doi.org/10.2307/3544207>
14. Kabos, W. J. (1964). VIII Tweevleugelige Insekten &#x02010; Diptera, De Nederlandse vliegen (Muscidae). In G. Houtman (Ed.). Wetenschappelijke Mededelingen van de Koninklijke Nederlandse Natuurhistorische Vereniging.(53, 1-32). Hoogwoud: Koninklijke Nederlandse Natuurhistorische Vereniging.
15. Kabos, W. J. (1975). Tweevleugelige Insekten &#x02010; Diptera, Nederlandse Vliegen &#x02013; Muscidae. In G. Houtman, &#x00026; H. D. van Bohemen (Eds.). Wetenschappelijke Mededelingen van de Koninklijke Nederlandse Natuurhistorische Vereniging.(110, 1-64). Hoogwoud: Koninklijke Nederlandse Natuurhistorische Vereniging.
16. Klecka, J., Hadrava, J., Biella, P., &#x00026; Akter, A. (2018). Flower visitation by hoverflies (Diptera: Syrphidae) in a temperate plant&#x02010;pollinator network. PeerJ, 6, e6025. <https://doi.org/10.7717/peerj.6025>
17. Krenn, H. W., &#x00026; Bauder, J.&#x02010;A.&#x02010;S. (2018). Morphological fine tuning of the feeding apparatus to proboscis length in Hesperiidae (Lepidoptera). Journal of Morphology, 279, 396-408. <https://doi.org/10.1002/jmor.20780>.
18. Kugler, H. (1970). Bl&#x000FC;ten&#x000F6;kologie. G. Fischer.
19. Loken, A. (1973). Studies on Scandinavian Bumble Bees (Hymenoptera; Apidae). Norsk entomologisk Tidsskrift. 20, .1-218.
20. Mauss, V., Treiber, R. &#x00026; Schmid&#x02010;Egger, C. (2004). Faltenwespen. Bestimmungsschl&#x000FC;ssel f&#x000FC;r die Faltenwespen (Hymenoptera: Masarinae, Polistinae, Vespidae und Eumenidae) der Bundesrepublik Deutschland. (3, 1-106). Deutscher Jugendbund f&#x000FC;r Naturbeobachtung (DJN).
21. Neuenkamp, L., Moora, M., &#x000D6;pik, M., Davison, J., Gerz, M., M&#x000E4;nnist&#x000F6;, M., Jairus, T., Vasar, M., &#x00026; Zobel, M. (2018). The role of plant mycorrhizal type and status in modulating the relationship between plant and arbuscular mycorrhizal fungal communities. New Phytologist, 220, 1236-1247. <https://doi.org/10.1111/nph.14995>.
22. Okamoto, T., Kawakita, A., &#x00026; Kato, M. et al (2008). Floral adaptations to nocturnal moth pollination in Diplomorpha (Thymelaeaceae). Plant Species Biology, 23, 192-201. <https://doi.org/10.1111/j.1442-1984.2008.00222.x>
23. Paulus, H. F., &#x00026; Krenn, H. W. (1996). Vergleichende Morphologie des Schmetterlingsr&#x000FC;ssels und seiner Sensillen&#x02013; Ein Beitrag zur phylogenetischen Systematik der Papilionoidea (Insecta, Lepidoptera). Journal of Zoological Systemetics and Evolutionary Research, 34, 203-216. <https://doi.org/10.1111/j.1439&#x02010;0469.1996.tb00826.x>
24. Sexton, R. (2014). The moth pollinators of greater butterfly orchids <i>Platanthera chlorantha</i> in Central Scotland. Journal of the Hardy Orchid Society, 11, 14-22.
25. Tschorsnig, H.&#x02010;P., &#x00026; Herting, B. (1994). Die Raupenfliegen (Diptera: Tachinidae) Mitteleropas: Bestimmungstabellen und Angaben zur Verbreitung und &#x000D6;kologie der einzelnen Arten. Serie A (Biologie) (1-170). Stuttgart: Stuttgarter Beitr&#x000E4;ge zur Naturkunde.
26. van Veen, M. P. (2009). Hoverflies of Northwest Europe: Identification keys to the Syrphidae. 2, KNNV Uitgeverij.
27. Witt, R. (1998). Wespen beobachten, bestimmen. Naturbuch&#x02010;Verlag.
